# Membrane‐Based Olefin/Paraffin Separations

**DOI:** 10.1002/advs.202001398

**Published:** 2020-08-09

**Authors:** Yanxiong Ren, Xu Liang, Haozhen Dou, Chumei Ye, Zheyuan Guo, Jianyu Wang, Yichang Pan, Hong Wu, Michael D. Guiver, Zhongyi Jiang

**Affiliations:** ^1^ Key Laboratory for Green Chemical Technology of Ministry of Education School of Chemical Engineering and Technology Tianjin University Tianjin 300072 P. R. China; ^2^ Collaborative Innovation Center of Chemical Science and Engineering (Tianjin) Tianjin 300072 P. R. China; ^3^ State Key Laboratory of Materials‐Oriented Chemical Engineering College of Chemical Engineering Nanjing Tech University Nanjing 210009 P. R. China; ^4^ State Key Laboratory of Engines School of Mechanical Engineering Tianjin University Tianjin 300072 P. R. China; ^5^ Joint School of National University of Singapore and Tianjin University International Campus of Tianjin University Binhai New City Fuzhou 350207 P. R. China

**Keywords:** carrier‐based membranes, channel‐based membranes, framework structures, network structures, olefin/paraffin separations, structure–performance relationships

## Abstract

Efficient olefin/paraffin separation is a grand challenge because of their similar molecular sizes and physical properties, and is also a priority in the modern chemical industry. Membrane separation technology has been demonstrated as a promising technology owing to its low energy consumption, mild operation conditions, tunability of membrane materials, as well as the integration of physical and chemical mechanisms. In this work, inspired by the physical mechanism of mass transport in channel proteins and the chemical mechanism of mass transport in carrier proteins, recent progress in channel‐based and carrier‐based membranes toward olefin/paraffin separations is summarized. Further, channel‐based membranes are categorized into membranes with network structures and with framework structures according to the morphology of channels. The separation mechanisms, separation performance, and membrane stability in channel‐based and carrier‐based membranes are elaborated. Future perspectives toward membrane‐based olefin/paraffin separation are proposed.

## Graphical abstract

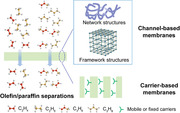

## 1 Introduction

Olefins, especially ethylene and propylene, have long been one of the most important chemical raw materials because of the broad application of their downstream products, including polyethylene, polypropylene, poly(vinyl chloride), and ethanol, since the chemical and petroleum industry began to develop in 1920s.^[^ ^1^ ^]^ The global production capacity of ethylene and propylene is 169 and 116 MMt per year, respectively, in 2017.^[^ ^2^ ^]^ Currently, the starting materials for olefin production consist of oil (naphtha and light paraffin), coal, and natural gas. The product of steam cracking of naphtha contains about 5 wt% of paraffin. For ethane cracking, which is more attractive because of shale gas development and lower cost, the product contains more than 40 wt% paraffin. In addition, when utilizing methanol and natural gas as the feedstock, the paraffin content ranges from 7–20 wt% depending on the catalyst and operation conditions. However, the downstream of the olefin industry needs high‐purity olefins. Monomer grade olefins require an ethylene purity of ≥99.9 wt% and a propylene purity of ≥99.5 wt% for polyolefin production.^[^ ^3^ ^]^ Chemical grade olefins require an olefin purity of ≥95 mol%. Therefore, highly efficient separation of ethylene/ethane and propylene/propane is in enormous demand in the chemical industry.

Nevertheless, it is a great challenge to separate olefins over paraffins with the same carbon numbers because of the closely similar physical properties between ethylene and ethane, as well as propylene and propane, as listed in **Table** **1**.^[^ ^4^ ^]^ Nowadays, the dominant technology for olefin/paraffin separation is cryogenic fractional distillation, which requires low temperature (as low as −90 °C), high pressure (usually 2 MPa), and a high reflux ratio (usually greater than 10), as well as more than 100 stages for the production of polymer‐grade olefins because of the low relative volatility for both ethylene/ethane and propylene/propane systems.^[^ ^5^ ^]^ Thus, olefin/paraffin separation imposes a heavy burden to global energy consumption and concurrent carbon dioxide emissions.^[^ ^6^ ^]^ Currently, purification of propylene and ethylene accounts for 0.3% of global energy consumption and is recognized as one of the seven chemical separations to change the world.^[^ ^7^ ^]^ With regard to the chemical properties, olefin has one distinct difference from paraffin in having a double bond in olefin and single bonds in paraffin. This difference in chemical property could be exploited to break the bottleneck of separation, which only relies on physical mechanism. The manipulation of both physical and chemical separation mechanisms to intensify the separation processes would also provide guidance on many other challenging separations. Therefore, the exploration of olefin/paraffin separation is not only important to energy and the environment, but of great significance for the sustainable development of separation science and technology.

**Table 1:** Physical properties of C_2_ and C_3_ olefin and paraffin

Component	Boiling point [K]	Critical temperature [K]	Kinetic diameter^a)^ [nm]	Polarizability × 10^25^ [cm]	Dipole moment × 10^18^ [esu cm]
Ethylene	169.5	282.3	0.423	42.5	0
Ethane	184.5	305.3	0.442	44.3	0
Propylene	225.5	364.9	0.468	62.6	0.366
Propane	231.1	369.8	0.506	63.3	0.084

a) Lennard–Jones diameter.

In the past three decades, olefin/paraffin separation has attracted increasing interest worldwide. Alternative process technologies, such as absorption and adsorption, have been developed. For the absorption and adsorption processes, subsequent desorption is required to obtain purified olefins, which imposes an unavoidable energy penalty in addition to increased process complexity.^[^ ^3a^ ^]^ Membrane technology is a forward‐looking technology for olefin/paraffin separations, which relies on the chemical potential difference from thermodynamic, kinetic, and molecular properties of olefin and paraffin molecules.^[^ ^8^ ^]^ Membrane separation can be conducted under mild conditions without phase change and can be accomplished in one step, which has the potential to significantly reduce energy consumption by up to 80% along with the associated carbon footprint.^[^ ^9^ ^]^ According to some analysis, membrane technology could save up to 0.3–1.5 GJ of energy per metric ton of olefin.^[^ ^7^ ^]^ It is reported that if gas selectivity is greater than 10, a membrane process can be coupled with distillation to achieve energy saving.^[^ ^10^ ^]^ When the selectivity is greater than 35, membrane technology can be applied independently toward olefin/paraffin separations.^[^ ^4b^ ^]^ In addition, it is more effective for energy savings in industrial separation processes by improving membrane selectivity than permeability.^[^ ^11^ ^]^ It is advised to focus on increasing membrane selectivity once a minimum permeance of 32 gas permeation unit (GPU) is obtained.^[^ ^12^ ^]^ Furthermore, membrane stability and mixed gas transport data are important considerations. Considering the rich diversity of membrane materials, preparation methods, modification strategies, and the flexible integration of physical and chemical mechanisms, membrane technology holds great promise as a process alternative to cryogenic distillation in olefin/paraffin separations.

The exploration of membrane technology toward olefin/paraffin separations often follows the sequence: membrane materials, preparation methods, membrane structures, separation mechanisms, and separation performance. Given the great separation challenges, developing new membrane materials/structures and manipulating the synergy of multiple separation mechanisms are among the core issues for membrane technology. Cell membranes found in nature provide excellent prototypes for highly efficient mass transport because of their precisely constructed structures and the integration of multiple separation mechanisms. There are two types of mass transport mechanisms through cell membranes. One is based on physical transport mechanism, usually mediated by channel proteins, which create a continuous pathway to convey specific solutes across the cell membrane. For example, aquaporins confer a fast transport passage for water molecules with a permeability of 3 × 10^9^ water molecules per subunit per second.^[^ ^13^ ^]^ Moreover, phospholipid bilayers create transient pathways for some gases, such as O_2_. These transient pathways can also be regarded as channels. The other is based on chemical transport mechanism, mediated by carrier proteins, which impart numerous chemical binding sites with solutes and facilitate the transmembrane permeation. For instance, the active transport of Na^+^ and K^+^ across cell membranes via the sodium–potassium pump is just through specific carrier proteins.

**Figure 1:**
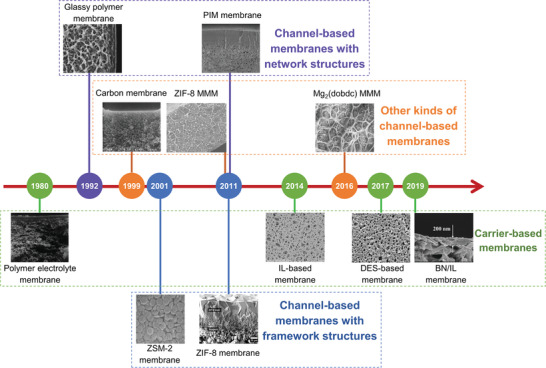
The roadmap of membrane materials development for olefin/paraffin separations. The image of glassy polymer membrane was reproduced with permission.^[^ ^14^ ^]^ Copyright 2002, Elsevier. The image of PIM membrane was reproduced with permission.^[^ ^15^ ^]^ Copyright 2017, Elsevier. The image of ZSM‐2 membrane was reproduced with permission.^[^ ^16^ ^]^ Copyright 2001, Elsevier. The image of ZIF‐8 membrane was reproduced with permission.^[^ ^17^ ^]^ Copyright 2010, Elsevier. The image of polymer electrolyte membrane was reproduced with permission.^[^ ^18^ ^]^ Copyright 2001, Elsevier. The image of IL‐based membrane was reproduced with permission.^[^ ^19^ ^]^ Copyright 2018, Elsevier. The image of DES‐based membrane was reproduced with permission.^[^ ^20^ ^]^ Copyright 2017, American Chemical Society. The image of BN/IL membrane was reproduced with permission.^[^ ^21^ ^]^ Copyright 2019, John Wiley and Sons. The image of ZIF‐8 MMM was reproduced with permission.^[^ ^22^ ^]^ Copyright 2011, Elsevier. The image of Mg_2_(dobdc) MMM was reproduced with permission.^[^ ^23^ ^]^ Copyright 2016, Springer Nature. The image of carbon membrane was reproduced with permission.^[^ ^24^ ^]^ Copyright 1999, American Chemical Society.

So far, various membrane materials have been explored toward olefin/paraffin separations, including polymeric membranes,^[^ ^14^, ^15^ ^]^ zeolite membranes,^[^ ^16^ ^]^ metal organic framework (MOF) membranes,^[^ ^17^ ^]^ facilitated transport membranes,^[^ ^18^, ^19^, ^20^, ^21^ ^]^ mixed matrix membranes, and carbon membranes (**Figure** **1**).^[^ ^22^, ^23^, ^24^ ^]^ However, it remains very challenging for synthetic membranes to achieve simultaneously high permeance and high selectivity. Highly efficient mass transport in cell membrane structures shows that channels and carriers are key to achieve both high permeance and high selectivity. These structures could be extended to synthetic membranes and thus we present for the first time an overview on “membrane‐based olefin/paraffin separations” from the viewpoint of channel‐based and carrier‐based membranes. In other words, membranes for olefin/paraffin separations are categorized into channel‐based membranes through physical separation mechanisms and carrier‐based membranes through chemical separation mechanisms.

**Figure 2:**
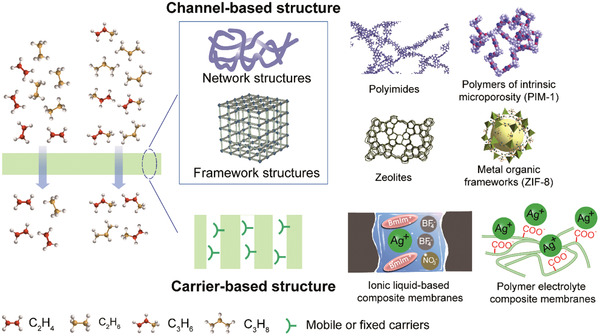
Schematic of channel‐based and carrier‐based membranes toward olefin/paraffin separations.

In this review, we first elaborate on channel‐based and carrier‐based membrane materials; then channel‐based membranes are further subdivided into those with network structures and framework structures (**Figure** **2**). The manipulation of physical and chemical microenvironments within channel‐based and carrier‐based membranes is highlighted. Second, the physical and chemical mechanisms toward olefin/paraffin separations are elucidated in depth. The enthalpic/entropic selectivity and the synergistic regulation of multiple separation mechanisms are emphasized. Third, the applications of channel‐based and carrier‐based membranes toward ethylene/ethane and propylene/propane gas pairs are summarized, respectively. Representative membranes in these two different separation systems are analyzed. Further, the membrane stability, including the deactivation of facilitated transport carriers, plasticization effects, and aging effects, is discussed. This review aims to offer guidance on the rational design of high‐performance olefin/paraffin separation membranes.

## 2 Membranes: Chemistries, Materials, and Structures

### 2.1 Channel‐Based Membranes

The concept of “channel‐based membranes” discussed here refers to membranes wherein the transport of molecules is based on passages without chemical interactions with membrane materials. The passage includes free volumes in most polymeric membranes and micropores in some microporous polymeric membranes, organic–inorganic hybrid membranes, and most inorganic membranes. Free volume distributes in the polymer matrix where polymer chains interlace, entangle, and form a network structure. The micropores in microporous polymers are random and disordered. This might be analogous to transient and random pathways in lipid bilayers, and will be affected by transient polymer chain motions in the networks. In contrast, the micropores in zeolites and MOFs are distributed along regular frameworks, which are formed by the growth of building blocks with ordered, periodic, and interconnected porous structure. This could be analogous to channel proteins, where micropores are permanent and highly efficient mass transport pathways. Therefore, for the first time, we further classify channel‐based membranes into those with network structures and those with framework structures according to the morphology of the channels. Representative examples of both are elucidated and their structures are highlighted below.

#### 2.1.1 Membranes with Network Structures

Network structure in this article refers to polymeric membranes having random order and amorphous channels. In general, a typical network domain is formed by the disordered stacking and interweaving of flexible units. Thus, most olefin/paraffin separation membranes with network structures are polymeric, both rubbery and glassy polymers.^[^ ^25^ ^]^ The channels are formed due to random thermal motions and irregular polymer chain packing. Before around 2000, most work focused on exploring different rubbery or glassy polymers to achieve high separation performance.^[^ ^26^ ^]^ Among them, various polyimide (PI) membranes appeared to have the most promising performance. After 2000, more attention was turned to solving plasticization and aging effects by means of crosslinking and annealing treatment.^[^ ^14^, ^27^ ^]^ Meanwhile, in 2004, a new class of polymer, called polymers of intrinsic microporosity (PIMs), emerged.^[^ ^28^ ^]^

##### Channels in Network Structures

An important parameter of network structure is the fractional free volume (*FFV*), which arises from the different sized interchain voids randomly distributed in polymers, providing space for segmental chain motion and mass transport.^[^ ^29^ ^]^ *FFV* is calculated using Equation (1)

(1) $$\mathrm{FFV}=\left(V_{T}\;-\;V_{0}\right)/V_{T}$$

where *V*_T_ is the specific volume of polymer at temperature *T* (298 K), *V*_0_ is the hypothetical volume at 0 K, which is estimated from Equation (2) with the van der Waals volume (*V*_w_) of Bondi^[^ ^30^ ^]^

(2) $$V_{0}=1.3V_{W}$$

Besides *FFV*, Guo has developed a theory to distinguish different types of free‐volume in materials.^[^ ^31^ ^]^ The non‐collapsible free volume “void” based on molecular configuration, rather than conformation, is called internal free volume. The configuration‐based free volume is intrinsic, similar to those present in inorganic molecular sieve materials. The intrinsic cavity formed by triptycene unit geometry is a typical example. The conformation‐based free volume is subject to constant changes, depending on chain dynamics. Here, we extend this theory to channels in network structures, which can be treated as consisting of both configuration‐based and conformation‐based free volume. Rubbery polymers and glassy polymers possess different proportions of these two types of free volumes. Thus, the properties of channels are different in rubbery polymers and glassy polymers. Besides, glass transition temperature (*T*_g_) is also a parameter reflecting channel flexibility. A high *T*_g_ means higher energy is required for chain movement, that is, lower channel flexibility.

##### Network Structures in Conventional Polymeric Membranes without Intrinsic Micropores

**Figure 3:**
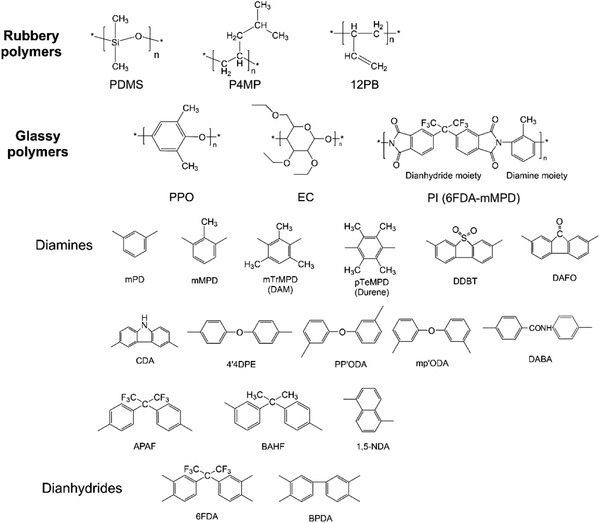
Chemical structures of rubbery and glassy polymers, as well as the diamine and dianhydride moieties of polyimides.

Herein, conventional polymeric membranes refer to those without intrinsic micropores. These membrane materials are sub‐divided into rubbery polymers and glassy polymers based on the *T*_g_ (**Figure** **3**). Macroscopically, rubbery polymers exhibit low *T*_g_ with higher failure strain and are in the rubbery state at ambient temperature. Some rubbery polymeric membranes, including poly(dimethylsiloxane) (PDMS), poly(4‐methyl‐1‐pentene) (P4MP), and 1,2‐polybutadiene (12 PB) membranes, have been summarized by Okamato et al. for the separation of propane/propylene.^[^ ^26b^ ^]^ Microscopically, the amorphous network is formed by polymer chain entanglement with weak interchain interactions and considerable chain reformation. The energy barrier of single bond rotation is usually very low, such as Δ*E*(H_3_Si‐SiH_3_) = 4.2 kJ mol^−1^ and Δ*E*(CH_3_‒SiH_3_) = 7.1 kJ mol^−1^, allowing easy polymer segmental conformational changes. Thus, channels in rubbery polymeric membranes are mostly composed of conformation‐based free volume, which is flexible and transient. Rubbery polymers often have large *FFV* (more than 20%), which accounts for the observed high membrane gas permeability.^[^ ^25b^ ^]^

In glassy polymers, the *T*_g_ is higher than ambient temperature and thus polymer chain mobility is inhibited at ambient temperature. Among various glassy polymers, polyimides are the most widely investigated in olefin/paraffin membrane separation. Polyimides are obtained by the condensation reaction between dianhydride monomers and diamine monomers. The monomer structure determines the configuration of polymer chains, the interchain interactions, stacking of polymer chains, and further, the network structure in the polyimide membranes. Therefore, various monomers have been investigated through regulating the twist structure of the main chains, the rigidity and flexibility of main chains, as well as substituents in side chains. By incorporating different groups into the main chains and side chains of polyimides, the rigidity can be regulated by segmental torsion and steric hindrance. Polyimides containing large aromatic structures in the main chains usually exhibit more configuration‐based free volume because the rigid aromatic rings in the network structure hinder segmental motion and dense packing of the polymer chains. In contrast, flexible segments such as ether linkages in the main chains lower *T*_g_, enhance stacking efficiency, and increase cohesive energy density and conformation‐based free volume, as shown in **Table** **2**.^[^ ^36^ ^]^ Moreover, the introduction of conjugate structures into main chains will cause the polymer chain conformation to twist, creating more voids and increasing *FFV*. For example, the *FFV* of DDBT‐based polyimides is larger than that of mPD‐based polyimides. For DDBT‐based polyimides, the ring inhibits internal rotation around the C‒N bond and results in a nonplanar structure. The neighboring dibenzothiophene‐5,5‐dioxide and imide rings are almost perpendicular to each other, leading to loose packing of polymer chains.^[^ ^34^ ^]^ Besides, the steric effect of bulky groups in side chains also affect the formation of the network structure. For example, methyl substitution on the phenyl ring in a diamine moiety tends to increase the *FFV* of polyimide membranes because of the inefficient packing of polymer chains. Furthermore, the *T*_g_ is increased due to higher chain rigidity from the introduction of methyl groups.^[^ ^32^ ^]^

**Table 2:** Physical properties of polyimides

Dianhydrides	Diamines	*T_g_* [K]	*ρ* ^a)^	CED [MJ m^−3^]	*FFV* [%]	Ref.
6FDA	mPD	548	1.478	767	15.8	[27a]
	mMPD	608	1.416	740	17.6	[32]
	mTrMPD	649	1.340	499	18.9	
	pTeMPD	679	1.325	487	18.5	
	44′DPE	564	1.434	536	16.2	[27a]
	mp′ODA	533	1.438	740	16.2	[33]
	pp′‐ODA	516	1.432	740	16.5	
	CDA	685	1.445	864	15.5	[34]
	DAFO	649	1.457	876	15.4	
	DDBT	>763	1.421	830	16.9	
	APAP	533	1.361	666	16.3	
	BAHF	578	1.482	576	18.2	[33]
	15′NDA	551	1.428	546	13.3	[27, 35]
	NDA/Durene (75:25)	—	1.399	—	14.8	[35]
	NDA/Durene (50:50)	—	1.363	—	16.7	
	NDA/Durene (25:75)	—	1.332	—	18.2	
BPDA	mTrMPD	658	1.241	999	15.5	[32]
	pp′‐ODA	543	1.366	1043	12.1	[33]
	CDA	630	1.379	864	15.4	[34]
	DDBT	>763	1.372	829	12.5	
	BAHF	602	1.424	740	15.7	[33]

a) Density [kg m^−3^].

Polar groups and charge transfer complexes can also regulate the network structure and channels in the membranes. Polyimides with higher fluorine content exhibit lower cohesive energy density (*δ*) values, indicating that fluorine reduces interchain interactions of network segments and polymer chain packing density. Tanaka et al. observed that 6FDA‐based polyimides had higher *FFV* than BPDA‐based polyimides.^[^ ^33^ ^]^ For polyimides, charge transfer complexes could be formed during the condensation reaction of a diamine moiety as an electron donor and a dianhydride moiety as an electron acceptor. Chan et al. showed that co‐polyimides from 6FDA and 1,5‐NDA (naphthalene)/durene diamines were found to have a higher packing density and lower FFV in the co‐polyimides with higher 6FDA‐NDA content due to the formation of more charge transfer complexes.^[^ ^35^ ^]^ The naphthalene diamine moiety of 6FDA‐NDA actively promoted electron transfer between donors and acceptors.

##### Network Structures in PIM Membranes

**Figure 4:**
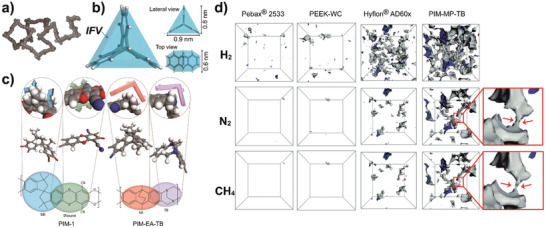
a) Space‐filling molecular model of a fragment of PIM‐1, showing its rigid, randomly contorted structure. Reproduced with permission.^[^ ^37^ ^]^ Copyright 2004, Royal Society of Chemistry. b) 3D visual of a triptycene building block, presenting its structurally fixed free volume elements and dimensions provided based on molecular dynamic simulation. Reproduced under the terms of the Creative Commons Attribution 4.0 International License.^[^ ^38^ ^]^ Copyright 2019, the Authors, Published by MDPI, Basel, Switzerland. c) Comparison of torsional rigidity of PIM‐1 and PIM‐EA‐TB. Reproduced with permission.^[^ ^39^ ^]^ Copyright 2013, American Association for the Advancement of Science. d) Molecular simulations showing the accessible free volume for H_2_, N_2_, and CH_4_ in Pebax2533, PEEK‐WC, HyflonAD60x, and PIM‐MP‐TB. Reproduced with permission.^[^ ^40^ ^]^ Copyright 2019, Royal Society of Chemistry.

PIMs (**Figure** **4**a) are a subclass of microporous polymers with rigid and contorted backbone structures, first reported by Budd and McKeown et al. in 2004.^[^ ^37^ ^]^ PIM‐1, the archetypal PIM, is synthesized from 5,5′,6,6′‐tetrahydroxy‐3,3,3′,3′‐tetramethyl‐1,1′‐spirobisindane (TTSBI) and 2,3,5,6‐tetrafluorotephthalonitrile (TFTPN). The ladder‐like structure of the main chain and the spirocyclic‐centers in the SBI increase the rigidity of the polymer chains, which pack inefficiently, thereby increasing the fraction of configuration‐based free volume and creating microporous network channels. The polymer backbone has a twisted ladder‐like structure, which resists rotation, resulting in a more uniform pore structure.^[^ ^39^, ^41^ ^]^ As a result, PIM‐1 exhibits a *T*_g_ of up to 715 K and *FFV* of ≥22%.^[^ ^42^ ^]^ Khan et al. have applied PIM‐1 to C_3_H_6_/C_3_H_8_ membrane separation.^[^ ^28a^ ^]^ The separation performance surpassed the upper bound of C_3_H_6_/C_3_H_8_ system defined in 2003. Apart from PIM‐1, a series of microporous polyimides prepared with rigid triptycene‐based diamine monomers, such as KAUST‐PI‐1, are highly shape‐resistant.^[^ ^28^, ^38^, ^43^ ^]^ The introduction of triptycene into PIM results in higher internal free volume (IFV) (Figure 4b). These structural units are composed of fused‐ring ladder‐like structures, which further reduce chain mobility (Figure 4c).

Fuoco et al. provided a visual illustration of channels in different types of polymeric membranes, including rubbery Pebax 2533, glassy PEEK‐WC, Hyflon AD60x, and PIM‐MP‐TB, by optimized chain‐packing models.^[^ ^40^ ^]^ This model informs which free volume elements can be utilized by H_2_, N_2_, and CH_4_ (Figure 4d). In detail, the model directly infers that the gas diffusion channels in rubbery polymers are instantaneous rather than permanent. In contrast, glassy polymers have more configuration‐based diffusion channels. As the size of gas molecules increases, the interconnected diffusion volume in the model starts to reduce, leading to a decrease in the diffusion rate. PIM‐MP‐TB has the highest diffusion volume and interconnected diffusion channels.

In this section, we have provided an overview of the network structures in polymeric membranes, especially free volume elements, which function as transport pathways. In rubbery polymeric membranes, the dominant conformation‐based free volume is flexible and poor at distinguishing between olefins and paraffins. Thus, these membranes exhibit poor olefin/paraffin selectivity. Therefore, membrane materials with more configuration‐based transport channels and certain chain‐segmental rigidity, such as polyimides and PIMs, are expected to exhibit better separation performance.

#### 2.1.2 Membranes with Framework Structures

Different from membranes with network structures, membranes with framework structures feature permanently ordered gas transport channels, which originate from the interconnected micropores. The key parameters of membranes with channel structures come from the pore architectures and grain boundaries. Pore architectures refer mainly to pore size, pore size distribution, and pore surface chemical properties. In the past few years, major efforts have been devoted to fabrication and manipulation of membranes with framework structures toward olefin/paraffin separations. Zeolite and MOF materials, as well as the strategies to manipulate pore architectures and grain boundary structures are highlighted below.

##### Zeolite Membranes

**Figure 5:**
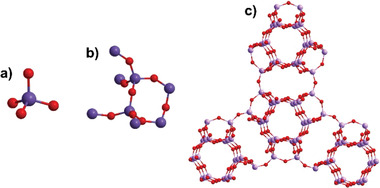
a) Primary building blocks of [SiO_4_]^4−^ tetrahedron, b) secondary building blocks formed by the combination of primary building blocks with a specific spatial arrangement, and c) the framework structure of ZSM‐5 produced by the linkage of secondary building blocks with a unique system of channels and cages. The red and purple in the framework represent oxygen and silicon atoms, respectively, and the aluminium elements are not shown.

Zeolites are crystalline microporous materials with infinitely extended 3D interconnected framework architecture (**Figure** **5**). The framework of zeolites mainly consists of SiO_4_ and AlO_4_ tetrahedra covalently bonded by shared O atoms. The AlO_4_ units bear negative charges, which are balanced by specific cations in the pores. Zeolite architecture possesses abundant channels, cavities, or pores. The uniform pore sizes on a molecular scale (0.3–0.7 nm) endow zeolites with molecular sieving ability. In addition, the cations within pores can be exchanged into other ions. The rigid framework structure and molecular sieving capability make zeolites promising materials for the separation of mixtures with similar molecular sizes, including olefin/paraffin separations. There have been many reports about zeolite‐based adsorption processes for olefin/paraffin separations. In particular, Bereciartua et al. fabricated heart‐shaped zeolite, ITQ‐55, with controlled flexibility and pore chemistry, which allowed the separation of ethylene and ethane kinetically, with an ethylene/ethane selectivity more than 100.^[^ ^44^ ^]^ However, the application of zeolites in membrane‐based olefin/paraffin separation has been limited. The first report of the use of zeolite membranes for olefin/paraffin separation was a faujasite‐type Na‐X reported by Nikolakis et al. in 2001.^[^ ^16^ ^]^ In the same year, Pan et al. fabricated template‐free MFI‐type zeolite membranes and reported the separation of hydrocarbon species.^[^ ^45^ ^]^ Then, Giannakopoulos et al. conducted a more detailed investigation into faujasite‐type zeolite membranes toward propylene/propane separation in 2005.^[^ ^46^ ^]^ Several subsequent studies sought to improve zeolite membrane performance, including titanium‐containing zeolite (ETS‐10) membranes and Ag‐exchanged zeolite membranes.^[^ ^47^ ^]^

Zeolite membranes are usually fabricated on a ceramic support through in situ or secondary growth methods, which have been elucidated in other reviews in detail.^[^ ^48^ ^]^ A template is normally used when preparing zeolite membranes. The shape and size of templates are closely related to the framework structure of the target zeolite. Also, the template serves as the structure‐directing agent, induces the formation of specific framework structure, and improves the stability of the framework through host–guest interaction. However, the template still occupies the pores of zeolite membranes after synthesis and needs to be removed by calcination (up to 500 °C), which may induce intercrystalline gaps. Therefore, both zeolite micropores and intercrystalline gaps are transport channels for molecules.

Zeolite micropores are determined by the crystal structure. So far, several types of zeolites have been explored in olefin/paraffin separations, and the pore sizes vary for different zeolites, such as 7.4 Å for Na‐X,^[^ ^49^ ^]^ 5.5 Å for ZSM‐5, and 7.2 Å for ETS‐10.^[^ ^50^ ^]^ Zeolite micropore size can also be regulated by post‐synthesis modification; although no pore‐modified zeolite membranes have yet been applied to olefin/paraffin separations, some insights can be gained from this approach in other separation systems and zeolite‐based adsorption processes. First, cation exchange changes to the pore size. For example, 3A and 5A molecular sieves are obtained by the cation exchange of NaA zeolite with potassium and calcium ions, respectively. Second, chemical treatment of zeolite crystals can regulate the pore size. It is relatively easy to form a deposition layer on the external surface of zeolites to reduce the accessible pore size. Chemical vapor deposition of tetra(methoxy)silane has been utilized to deposit a uniform SiO_2_ layer.^[^ ^51^ ^]^ Also, Chudasama et al. reported a liquid‐phase deposition strategy by reaction of zeolites with silane in toluene, followed by the removal of solvent and calcination in air.^[^ ^52^ ^]^ However, it should be more effective, yet technically more difficult, to form a deposition layer within the micropores to reduce the pore diameter. Masuda et al. proposed that catalytic cracking of silane could be applied to deposit SiO_2_ layer within the micropores of MFI‐type zeolite.^[^ ^53^ ^]^ The silane was introduced and chemically adsorbed in the micropores, followed by catalytic cracking by the active sites of zeolites at 550 °C under N_2_, generating coke‐containing Si atoms. Subsequently, the resulting material was calcinated at 550 °C in air to form a SiO_2_ monolayer on active sites.

Intercrystalline gaps play different roles with the variation of their dimension. When the size of intercrystalline gaps is smaller than zeolite pores, the latter will dominate the membrane performance and the performance can be deduced by the zeolite crystal structures. When the size of intercrystalline gaps is larger than zeolite pores, the effect of intercrystalline gaps must be taken into account and the separation performance cannot be predicted merely based on the zeolite crystal structures. In more extreme cases, when the intercrystalline gaps are much larger than the intrinsic pores, these gaps will dominate membrane performance and act like defects, which nullify the molecular sieving effect of zeolite crystals. Therefore, efforts have been made to reduce or eliminate intercrystalline gaps, including template‐free secondary growth without calcination steps,^[^ ^54^ ^]^ rapid temperature processing during calcination,^[^ ^55^ ^]^ post‐treatment by blocking the intercrystalline regions with specific impregnation species, or counter‐diffusion chemical vapor deposition of silica precursors.^[^ ^56^ ^]^ Even though these methods are effective to decrease the size of intercrystalline gaps, the permeance of corresponding zeolite membranes is frequently compromised.

##### MOF Membranes

MOFs, similar to zeolites, are a class of microporous crystalline substances with high porosity (up to 90%).^[^ ^57^ ^]^ MOFs are constructed by ordered and infinitely extended coordinated interconnections between metal ions or metal‐containing clusters (serving as nodes) and multidentate organic ligands (serving as struts). Therefore, MOFs are a class of inorganic–organic hybrid materials. The variability of metal ions and organic linkers endows MOFs with almost infinite connection and topology possibilities. The pore structure of MOFs is in the molecular dimension and it can be precisely manipulated to exhibit a molecular sieving effect by designing metal ions/clusters, organic linkers, and topology. The high porosity, high specific surface areas (even beyond 6000 m^2^ g^−1^), interconnected transport channels endowed by the framework structure, and the molecular sieving effect make MOFs promising as adsorbents or membrane materials for gas separation, especially for the separation of similar‐sized molecules, such as xylene isomers and olefin/paraffin gas pairs.^[^ ^3^, ^58^ ^]^ Even though MOFs appear to have many merits for olefin/paraffin separations, only a few kinds of MOF membranes (such as ZIF‐8 and ZIF‐67 membranes) have been demonstrated to be effective for propylene/propane separation and none could separate ethylene/ethane effectively.

**Figure 6:**
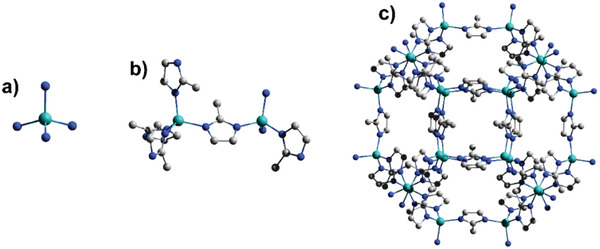
In ZIF‐8, the zinc ions are connected by MeIM ligands with coordinated bonding. a,b) The primary units of zinc–nitrogen tetrahedron expand to building blocks with a specific topology by sharing the same MeIM ligand between two adjacent zinc ions. c) The building blocks expand further to the framework structure with a unique system of channels and cages. All hydrogen atoms have been omitted for clarity. The cyan, blue, and grey in the structure represent the atoms of zinc, nitrogen, and carbon, respectively.

ZIF‐8, with a sodalite‐topology and structure constructed by zinc ion nodes and 2‐methylimidazole (MeIM) ligands (**Figure** **6**), is the most studied MOF for propylene/propane separation. Even though the crystallographic pore aperture of ZIF‐8 is 3.4 Å, it can uptake guest molecules as large as about 7.6 Å (1,2,4‐trimethylbenzene) because of the swing effect of the organic linker or the lattice flexibility.^[^ ^59^ ^]^ Thus, ZIF‐8 membranes exhibit low selectivity of about only 5 for CO_2_/CH_4_ gas pairs (3.3 and 3.8 Å, respectively).^[^ ^60^ ^]^ However, ZIF‐8 membranes exhibit a sharp cut‐off between propylene (about 4.0 Å) and propane (about 4.3 Å). Thus, the effective pore size of ZIF‐8 is regarded as 4.0 Å.^[^ ^61^ ^]^ In this case, the framework flexibility contributes to the efficient separation of propylene and propane.

For ZIF‐8 membranes, exploration on preparation methods is also one research focus in order to meet the requirements of industrial application. So far, various methods have been reported, including secondary growth, counter‐diffusion, interfacial microfluidic membrane processing (IMMP), electrochemical deposition and vapor deposition. Compared with zeolite membranes, templates are not necessary for MOF membrane preparation and the synthesis conditions do not require high temperatures. The coordination property between two compounds endows the framework construction of MOF membranes with more flexibility.

**Figure 7:**
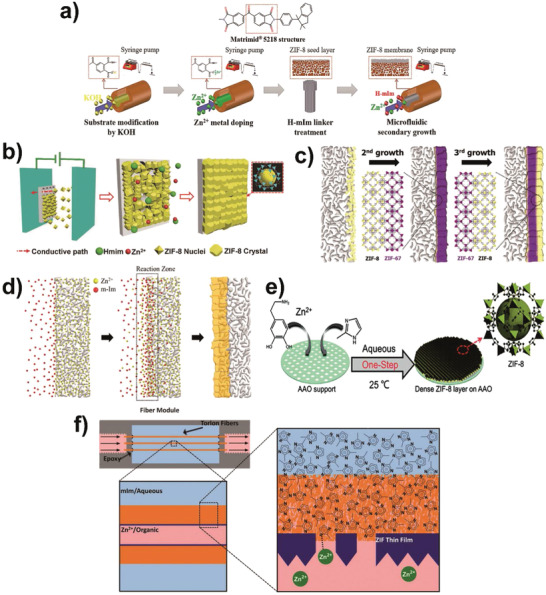
Preparation methods for ZIF‐8 membranes. Secondary growth method: a) the synthesis of ZIF‐8 hollow fiber membranes by combining the modification of polymer supports to optimize the seeding layer structure and microfluidic secondary growth. Reproduced with permission.^[^ ^62^ ^]^ Copyright 2019, American Chemical Society. b) The synthesis of ZIF‐8 membranes by an electrophoretic process to assemble ZIF‐8 seed crystals on the support and followed by secondary growth in the synthesis sol for a short period of time. Reproduced with permission.^[^ ^63^ ^]^ Copyright 2018, John Wiley and Sons. c) Hetero‐epitaxial growth of the ZIF membrane with two different layers (ZIF‐8 and ZIF‐67) on ZIF‐8 seed crystals. Reproduced with permission.^[^ ^64^ ^]^ Copyright 2015, American Chemical Society. d) The synthesis of ZIF‐8 membrane by the counter‐diffusion method. The support saturated with a metal precursor solution is placed in a ligand solution containing sodium formate. The diffusion of metal ions and ligand molecules cause the formation of a “reaction zone” at the interface. Rapid heterogeneous nucleation/crystal growth in the vicinity at the interface leads to continuous well‐intergrown ZIF‐8 membranes. Reproduced with permission.^[^ ^65^ ^]^ Copyright 2013, American Chemical Society. e) ZIF‐8 membrane prepared by dopamine‐mediated counter‐diffusion method. Dopamine was added into the metal ion solution, which functions as a competitive ligand to MeIM, chelating Zn^2+^ ions in the solution. Also, dopamine polymerized on the support surface to anchor Zn^2+^ ions and MeIM promotes the counter‐diffusion growth of the ZIF‐8 layer on the support. Reproduced with permission.^[^ ^66^ ^]^ Copyright 2019, Royal Society of Chemistry. f) A scheme depicting the IMMP approach for ZIF‐8 membranes in hollow fibers. The Zn^2+^ ions are supplied in 1‐octanol solution (light red) flowing through the bore of the fiber, whereas the MeIM linkers are supplied on the outer shell side of the fiber in an aqueous solution (light blue). The two precursors react and form a polycrystalline ZIF‐8 layer (dark blue) on the inner surface of the fiber. Reproduced with permission.^[^ ^67^ ^]^ Copyright 2014, American Association for the Advancement of Science.

For secondary growth, nanosized ZIF‐8 crystals are typically pre‐coated onto the support as a seeding layer, which functions as a nucleation center. Then, ZIF‐8 crystal seeds grow into a dense and continuous membrane by hydro‐ or solvothermal synthesis. Therefore, secondary growth decouples nucleation and crystal growth and possesses more controllability. Achieving a high quality of seeding layer is of vital importance for membranes with well‐designed microstructures. First, strengthening the interaction between the seeding layer and support is effective to facilitate nucleation on the support. For example, the surface of Matrimid 5218 membrane was hydrolyzed to generate abundant carboxyl groups, which enhance the hydrophilicity and facilitate metal ion enrichment near the support surface (**Figure** **7**a).^[^ ^62^ ^]^ This modification of the polymeric support favors the growth of a continuous ZIF layer. Second, the introduction of external fields, such as electromagnetic and electric field, also contributed to the deposition of the seeding layer. A microwave‐assisted seeding process was first reported by Kwon et al. and a densely packed ZIF‐8 seed layer was formed within a couple of minutes because of a rapid local temperature increase under microwave irradiation.^[^ ^68^ ^]^ The seeding layer exhibited strong adhesion with the support possibly because of the formation of covalent bonds between ligands and Al_2_O_3_ support. An electrophoretic method was explored to assemble ZIF‐8 seed crystals on the unmodified support (Figure 7b).^[^ ^63^ ^]^ Electrophoretic nuclei need only one‐step to grow the seeding layer and can be extended to various unmodified supports and MOF species.

For secondary growth, it is relatively easy to control the membrane structure, including composition, thickness, orientation, and the reproducibility. By the same token, secondary growth is more complex. However, the merits of secondary growth should be highlighted, as indicated by the mounting research momentum using this method. By means of secondary growth, it is relatively easy to fabricate hybrid MOF membranes, which combine two (or more) kinds of metal ions or organic ligands and construct different transport channels in one membrane. Wang et al. fabricated zinc‐substituted ZIF‐67 membrane by using hybrid metal ion precursors (Zn^2+^ and Co^2+^) on the ZIF‐67 nanocrystal seeding layer.^[^ ^69^ ^]^ Also, membranes with two layers of different MOFs can be fabricated through secondary growth. This strategy usually requires similar crystalline topology. Kwon et al. explored hetero‐epitaxial growth of ZIF‐67 layer on ZIF‐8 seed crystals, and then another ZIF‐8 layer was grown onto the ZIF‐67 layer, as shown in Figure 7c.^[^ ^64^ ^]^ The interface between two MOF layers plays an important role in the membrane separation performance. Besides, secondary growth has the advantage of preparing MOF or zeolite membranes with oriented structure. Once the oriented seeding crystals are deposited, the orientation trend will be retained in the resulting membrane.

Both the counter‐diffusion method and IMMP can be regarded as an in situ technique. For ZIF‐8 membranes, the counter‐diffusion method was first proposed by Kwon et al. using inorganic support (Figure 7d) and then extended to polymer‐supported ZIF‐8 membranes.^[^ ^65^, ^70^ ^]^ The support was pre‐soaked in a metal ion solution and then transferred into the ligand solution to form thin‐layer MOF membranes through a solvothermal reaction. Furthermore, Jiang et al. reported a facile amelioration of the counter‐diffusion method through the introduction of dopamine to prepare ZIF‐8 membranes, as shown in Figure 7e.^[^ ^66^ ^]^ The dopamine serves as a modulator, which means that its self‐polymerization inhibits homogenous nucleation and slows down heterogeneous nucleation. Therefore, the ZIF‐8 nanocrystals could grow into large grains, which formed well‐intergrown dense membranes. IMMP was first reported by Brown et al. in 2014 for the preparation of ZIF‐8 hollow fiber membranes (Figure 7f).^[^ ^67^ ^]^ The metal ion and organic ligand solutions react at the interface of two solvents with controllable supply, replenishment, and recycling of reactants. Also, low‐cost polymers are utilized as the supports. Thus, IMMP is a promising approach for the scalable preparation of MOF‐based hollow fiber composite membranes.

**Figure 8:**
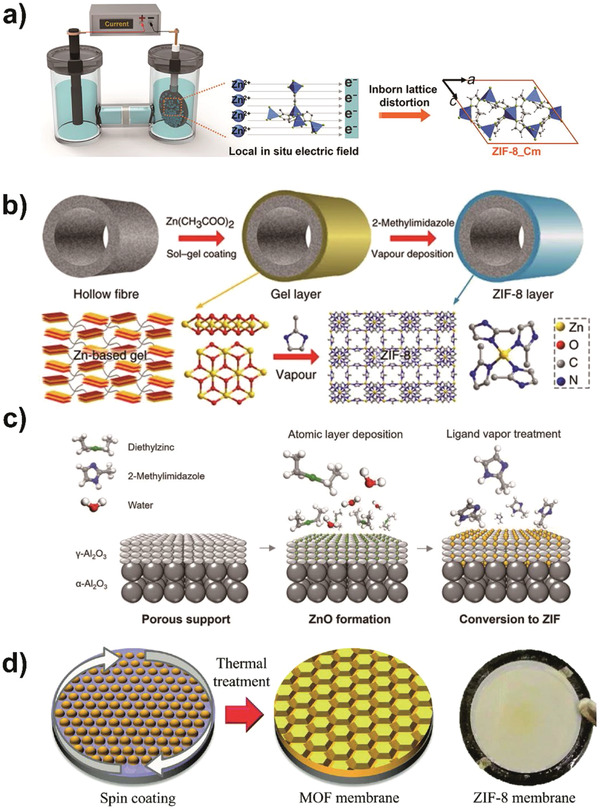
Emerging fabrication methods for ZIF‐8 membranes. a) An electrochemical cell for ZIF‐8 membrane growth by electrochemistry deposition, whereby the substrate serves as a cathode. With the local in situ electric field that forms around the support by the current, inborn lattice distortion occurs and a ZIF‐8 membrane with stiffer framework structures is formed. Reproduced with permission.^[^ ^71^ ^]^ Copyright 2018, American Association for the Advancement of Science. b) Schematic of ZIF‐8 membrane formation process by a gel–vapor deposition method. Zn‐based sol was coated on ammoniated poly(vinylidene fluoride) hollow fibers and transformed to Zn‐based gel by heat treatment. The MOF membrane was formed directly through ligand vapor deposition, whereby the produced vapor reacted with the sensitive Zn‐based gel and substituted organic chains to form more stable ZIF‐8. Reproduced under the terms of the Creative Commons Attribution 4.0 International License.^[^ ^73^ ^]^ Copyright 2017, Springer Nature. c) Schematic of an all‐vapor‐phase ligand‐induced permselectivation membrane fabrication process. The pores of the support are first blocked with ZnO through atomic layer deposition. The impermeable ZnO deposits are converted to permselective ZIF‐8 membrane by means of ligand–vapor treatment. Reproduced with permission.^[^ ^74^ ^]^ Copyright 2018, American Association for the Advancement of Science. d) Schematic of ZIF‐8 membrane synthesis by a two‐step sol–gel transformation, including gel coating for precursor dispersion and thermal treatment for crystallization. Reproduced with permission.^[^ ^75^ ^]^ Copyright 2018, Royal Society of Chemistry.

In order to further improve the controllability of preparation process, electrochemical and vapor deposition methods have been developed for ZIF‐8 membranes for olefin/paraffin separations. MOF membranes have been prepared by electrochemical deposition within several minutes under mild conditions (**Figure** **8**a). This approach used methanol as the solvent, needed electrolyte to improve the conductivity of the solution, and required only minor treatment to the support, but a conductive layer was required in the work of Zhou et al.^[^ ^71^ ^]^ Recently, Wei et al. further developed electrochemical deposition using water as the solvent without addition of any electrolyte or modulator.^[^ ^72^ ^]^ Vapor deposition is also a simple and controllable process, such as the gel–vapor deposition (GVD) method (Figure 8b).^[^ ^73^ ^]^ However, solvent cannot be avoided during the overall GVD process. Furthermore, Ma et al. reported for the first time the fabrication of ZIF‐8 membranes through an all‐vapor phase process, as shown in Figure 8c.^[^ ^74^ ^]^ A zinc oxide layer was first deposited onto the support by atomic layer deposition (ALD), followed by 2‐methyimidizole vapor‐treatment to convert the impermeable metal oxide layer to a selective MOF layer. The membrane structure could be manipulated by the number of ALD cycles. Simpler preparation methods have also been explored. Learning from polymeric membrane fabrication through solvent evaporation, Li et al. coated a gelatinous precursor dispersion onto a support, followed by thermal treatment for crystallization and membrane formation (Figure 8d).^[^ ^75^ ^]^ There was no need for support modification and the membrane thickness could be controlled by the gel concentration.

For ZIF‐8 membranes in olefin/paraffin separations, one of the goals is to improve selectivity further, because higher selectivity provides purer target olefins under the same stage‐cut and pressure ratio between the upstream and downstream sides of the membrane module. The manipulation of micropore structure is expected to improve the selectivity. Even though the framework flexibility endows ZIF‐8 membranes with high propylene/propane selectivity compared with other MOF and most conventional polymeric membranes, the flexibility is still too large that ZIF‐8 membrane cannot exhibit sharp molecular sieving between propylene and propane. Thus, the appropriate suppression of framework flexibility is needed to improve the selectivity further. Zhou et al. prepared ZIF‐8 membranes through a current‐driven method, with ZIF‐8_Cm being the major crystal structure whereby the ligand motion was limited and the framework was more rigid.^[^ ^71^ ^]^ Consequently, outstanding molecular sieving effect was achieved with propylene/propane selectivity of more than 300. Also, Sheng et al. found that a rubbery coating (such as the widely used PDMS coating) not only eliminated non‐selective intercrystalline boundary defects but also inhibited the ligand mobility and suppressed the framework flexibility of ZIF‐8.^[^ ^76^ ^]^

Apart from the crystalline structure of ZIF‐8, the grain boundary is another key factor that influences the reproducibility and separation performance of MOF membranes. Typically, PDMS coatings are convenient and effective in blocking intercrystalline defects.^[^ ^76^ ^]^ Also, a high nucleation density, which can be controlled by the preparation conditions, is beneficial for producing better grain boundary microstructure in MOF membranes. Lee et al. explored various morphologies of the seeding layer and suggested that a seed layer with a smaller grain size (about 100 nm) produced a better grain boundary than seeding grains of 1 µm.^[^ ^77^ ^]^ This is mainly because smaller seed crystals result in smaller grains, which are less intergrown and thus more favorable for eliminating intercrystalline defects. The ligand/node ratio also affects the nucleation density. Li et al. demonstrated that excess MeIM disturbed the crystallization process and formed large crystal grains, resulting in poor grain boundary microstructure in the case of too high MeIM/Zn ratio (about 6–12).^[^ ^75^ ^]^

#### 2.1.3 Other Kinds of Channel‐Based Membranes

Apart from membranes with network and framework structures, as well as carrier‐based membranes mentioned above, other kinds of membranes with channel structures, including mixed matrix membranes and carbon membranes, have been applied for olefin/paraffin separations.

##### Mixed Matrix Membranes

Mixed matrix membranes combine the processability of polymers and high selectivity of fillers. They generally comprise polymers as continuous matrix and highly selective fillers as dispersed phase. MOFs are the most widely used fillers for olefin/paraffin separations owing to their high porosity and micropore structures. However, it remains a challenge to construct ideal mixed matrix membranes with defect‐free interfacial structures and high separation performance. There are typically three kinds of transport channels in mixed matrix membranes, which are polymer matrix free volume, micropores in fillers, and interfacial channels or defects. There are no micropores for non‐porous fillers. For a specific polymer matrix, interfacial channels and fillers are the principal factors that need to be manipulated.

In many cases, interfacial channels or non‐selective defects should be avoided in mixed matrix membranes, to ensure that gas transport occurs through the porous fillers. Various kinds of interfacial channels typically form during membrane preparation. Especially with high filler loading, these channels may transform to defects, which are catastrophic to membrane performance. Interfacial defect formation can be alleviated to some extent by optimizing the membrane fabrication process. As illustrated by the work of Wu et al. and Adam et al., an optimized process is premixing part of the polymer solution by vigorously stirring it with the filler dispersion to stabilize the filler nanoparticles via the adsorption of polymer chains onto the particle.^[^ ^78^ ^]^ Then, the remaining polymer solution is added to prepare polymer/filler dispersion with the required concentration. In another approach, strengthening the interfacial interaction could also improve the compatibility between polymer matrix and filler nanoparticles, including modifying fillers with functional groups or alkyl chains. Ma et al. utilized hydrogen bonding between the polymer (6FDA‐DAM:DABA) and filler (MOF of UiO‐66‐NH_2_) to achieve stronger interfacial interaction and better compatibility.^[^ ^79^ ^]^ Likewise, introducing functional groups, such as ‒OH and ‒COOH, also contributes to improve interfacial structures and reduce defects. More detailed information about the interface in mixed matrix membranes is available in a review.^[^ ^80^ ^]^

For the exploration of fillers, there is a need to develop more advanced fillers, including new separation mechanisms or new structures. Liu et al. synthesized an MOF‐based filler with a triangular pore‐aperture, whose separation mechanism was demonstrated as a conformation‐dominated molecular sieving effect.^[^ ^81^ ^]^ Membrane pore structures with conformation‐based molecular sieving have rarely been explored, but this approach may provide new insights toward separating olefin/paraffin mixtures apart from dependencies on the difference of molecular size or chemical bonds. It is also promising to prepare mixed matrix membranes with both high filler loading (more than 50 wt%) and sufficient mechanical strength. Even though membranes with low filler loading (such as lower than 20 wt%) may exhibit better separation performance than pristine polymeric membranes, the overall performance is dominated by the properties of the polymer matrix. It is well known that some fillers exhibit excellent separation performance, such as ZIF‐8 membranes. This predicament can be solved by the concept of inverted phase mixed matrix membrane, i.e., from polymer‐dominated to filler‐dominated, to prepare membranes with much higher filler loading (even with filler as the matrix). However, this will require the solution to several problems, such as interfacial defects and poor mechanical properties, with increased filler loading. Strengthening matrix interactions and applying polymers with high molecular weight could help to achieve filler‐rich mixed matrix membranes. For example, Ma et al. fabricated ZIF‐8 dispersed polyimide membranes with a filler loading as high as 65 wt% through hydrogen bonding interactions between polymer chains (‒OH groups) and ZIF‐8 (N atoms).^[^ ^82^ ^]^

Besides, Rashidi et al. explored a class of atypical non‐polymeric mixed matrix membranes, wherein both the continuous phase (MOF of ZIF‐8) and filler phase (zeolite of MFI) were inorganic nanoporous crystalline materials.^[^ ^83^ ^]^ Compared with polymers, the nanoporous matrix exhibited two to three orders of magnitude higher permeability and one to two orders of magnitude higher selectivity. Thus, this all‐nanoporous hybrid strategy provides new directions for mixed matrix membranes.

##### Carbon Membranes

Carbon membranes are generally fabricated through pyrolysis of polymer precursors under controlled atmospheric and temperature conditions. The channels in carbon membranes manifest as micropores, which result from the imperfect packing of disordered, sp^2^‐hybridized, and condensed hexagonal carbon sheets under pyrolysis conditions. For olefin/paraffin separations, the polymer precursors of carbon membranes comprise phenolic resin,^[^ ^84^ ^]^ polyimides (such as Matrimid 5218 and 6FDA‐based species),^[^ ^85^ ^]^ polymers with an interpenetrating network, and PIMs (such as PIM‐1, PIM‐6FDA‐OH).^[^ ^86^ ^]^ The pores, or transport channels in CMS membranes, can be modeled by slit‐like structure and be described by a bimodal pore distribution of larger micropores (7–20 Å) interconnected by smaller ultramicropores (<7 Å).^[^ ^85c^ ^]^ The combination of rigid micropores and ultramicropores provides both high flux and high separation efficiency via molecular sieving, which will be elucidated in the “transport mechanism” section.

Since choosing appropriate polymer precursors is the first important step for preparing carbon membranes, the chemical structure and physical properties of polymer precursors should be primarily considered. First, the dimension of the polymer chains influences the packing of the graphitic carbon nanosheets under high pyrolysis temperatures. Precursors with more planar moieties tend to exhibit higher graphitization capability, which endows the carbon membranes with denser chain packing and a more ordered membrane structure. Second, bulky groups in the side chains weaken the coplanar configuration, thus preventing dense chain packing and hindering segmental motion, which results in relatively large free volume and loose chain packing membrane structure. For example, Xiao et al. compared four kinds of polyimide‐based carbon membranes having the same DAI diamine moiety (5,7‐diamino‐1,1,4,6‐tetramethylindan) but with different dianhydrides: 6FDA, BTDA, BPDA, and ODPA.^[^ ^87^ ^]^ The 6FDA‐based carbon membranes had the largest *FFV* because of the bulky ‒CF_3_ groups in the polymer backbone. Third, the thermal stability of polymer precursors influences the resulting carbon membrane structure. The open structure of carbon membranes is also the result of the evolution of gaseous products of precursor decomposition. Thus, carbon membranes derived from precursors with low thermal stability tend to possess more open structures and to be more gas permeable.

Pyrolysis conditions are also key factors for the channel construction of carbon membranes. Pyrolysis temperature, heating rate, pyrolysis atmosphere, and soaking time are the typical experimental variables. Generally, higher pyrolysis temperatures create more graphitized carbon sheets and a more ordered structures. During the graphitization process, some pore openings close or the pore size is reduced, with a narrower size distribution. Thus, higher pyrolysis temperatures usually lead to less gas‐permeable and more selective membranes. Second, at slow heating rates, the generation of evolved gas is slow and these molecules channel through the matrix in a more orderly way. The resulting micropore size is smaller and less favorable for the transport of large molecules. Therefore, a slow heating rate is more favorable for creating carbon membranes with a molecular sieving effect.^[^ ^88^ ^]^ Third, the pyrolysis atmosphere usually refers to vacuum, inert gas, or oxygen concentration. Vacuum conditions generally confer slightly smaller pore sizes and narrower pore size distribution compared with inert atmosphere.^[^ ^89^ ^]^ The oxygen concentration may change the pore structure remarkably; even a small amount of oxygen can activate carbon membranes. Oxygen molecules chemically adsorb on the micropores and form chemical bonds with carbon atoms, which reduce the micropore size and afford additional functional groups to carbon. Fourth, soaking time has a significant effect. Here, soaking time refers to the holding time at the maximum carbonization temperature during pyrolysis. A longer soaking time at relatively low temperature reduces the size of the micropore, resulting in the carbon membrane being less gas permeable and more selective. When the pyrolysis temperature is sufficiently high, soaking time will have less influence on the pore size and the temperature will be the determinant factor.

All these conditions need to be considered to design a suitable pyrolysis protocol. Chu et al. investigated an Fe‐containing polymer precursor comprising both 6FDA‐DAM:DABA (3:2) and Fe(acac)_2_.^[^ ^85a^ ^]^ They reported an optimized pyrolysis protocol by using a fast ramp rate of 10 °C min^−1^ and low pyrolysis temperature of 550 °C and obtained Fe‐containing carbon membranes with a specific oxidation state (Fe^2+^). Higher pyrolysis temperatures typically create more ordered pore structures, but because this can result in carbon membrane brittleness, a balanced approach to pyrolysis temperature is needed. Islam et al. used a relatively low temperature of 450 °C to fabricate carbon membranes with a combination of mechanical flexibility and micropore structure.^[^ ^90^ ^]^ The resulting carbon membrane could be bent into a U‐shape without fracturing. However, carbon membranes derived from the same precursor with a pyrolysis temperature of above 500 °C were brittle and unsuitable for gas permeation tests. Several other studies introduced additional compounds to the carbon membrane matrix, either to strengthen the interchain interaction, create crosslinking structure,^[^ ^86c^ ^]^ form micropores, or introduce specific interactions between the membrane matrix and target molecules.^[^ ^85^, ^86^ ^]^

### 2.2 Carrier‐Based Membranes

Carriers in the natural world have some common characteristics: (1) Biological carriers can only bind to specific components, which means that these carriers can only transport specific substances or very similar class of substances. (2) There exists saturated binding to biological carriers, which means that the binding sites are limited. (3) Physical and chemical microenvironments have a large impact on the function of biological proteins.

**Figure 9:**
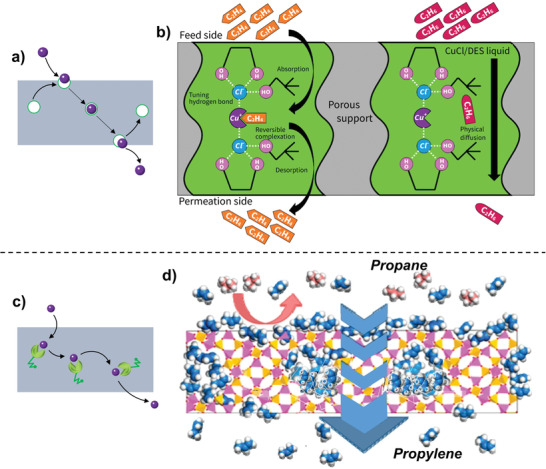
a) A representation of facilitated transport in mobile carrier membranes and b) corresponding application in a supported liquid membrane composed of CuCl as olefin carriers and deep eutectic solvents. Reproduced with permission.^[^ ^20^ ^]^ Copyright 2017, American Chemical Society. c) A representation of facilitated transport in fixed carrier membranes and d) corresponding application in an Ag‐exchanged Na‐X membrane for propylene/propane separation. Reproduced with permission.^[^ ^47c^ ^]^ Copyright 2019, American Chemical Society.

Inspired by biological carriers, herein, we summarize carrier‐based membranes for olefin/paraffin separations (**Figure** **9**). Carriers with specificity and saturation characteristics can be divided into two types: ions and nanoparticles. Transition metal salts (such as AgBF_4_, AgNO_3_, and CuCl) or metal nanoparticles are dispersed into the membrane matrix and interact with membrane materials, generating metal ions (Ag^+^ and Cu^+^), metal complex ions (CuCl_2_^−^), or nanoparticles with positively charged surfaces. These generated species serve as olefin carriers and facilitate the olefin transport in the membrane based on *π*‐complexation. The most researched carrier is Ag(I) ion. Besides, transition metal ions including Cu(I), Pd(II), Ru(III), and Ir(III) have also been investigated for olefin/paraffin separations.^[^ ^91^ ^]^ The Pd(II)‐ethylene complex was first recognized in 1827.^[^ ^92^ ^]^ Cu(I) is considered to be the key structure for the transmembrane sensor domain in the ethylene receptor proteins, such as ethylene response 1 (ETR1), in plants.^[^ ^93^ ^]^ Metal nanoparticles (Ag, Au, and Cu) have also been investigated as carriers for olefin/paraffin separations.^[^ ^94^ ^]^ Generally, compared with transition metal ions, these nanoparticles exhibit poorer activity, whereas their stability is better.^[^ ^95^ ^]^

Similar to biological systems, the carrier activity will be affected by the surrounding physical/chemical microenvironments. The physical microenvironment refers mainly to the dispersed matrix of the carrier, which determines the mobility of carriers. The chemical microenvironment determines the chemical activity of carriers.

#### 2.2.1 Dispersed Matrix of Carriers

The membrane matrix into which carriers are dispersed can be classified as liquid state and solid state. Membranes containing liquid dispersed matrix for carriers are liquid membranes and represented by supported liquid membranes (SLMs). In a typical SLM, the carriers are highly mobile in the liquid phase, and the liquid is generally entrapped within the pores of porous membranes, which are mainly polymer‐based materials. When an olefin molecule contacts the membrane on the upstream side, the carrier reversibly reacts with it and forms a complex. The carrier–olefin complex moves to the downstream side of the membrane under a concentration gradient and then dissociates into an olefin molecule and carrier by reversible reaction. The carrier continues to move from the downstream to the upstream under a concentration gradient to complete a carry cycle. In this microenvironment, carriers are also called mobile carriers. SLMs evolved from gas–liquid membrane contactors and emulsion liquid membrane in earlier years. Ward reported a similar structure containing a layer of porous material impregnated with a liquid film in 1972.^[^ ^96^ ^]^ With further investigations of liquid microenvironments, stable and nonvolatile ionic liquids (ILs) have attracted attention.^[^ ^19^, ^97^ ^]^ Also, the interaction between ILs and transition metal ions may contribute to the stability of carriers. For example, Dou et al. dispersed AgNO_3_ into protic ILs and achieved long‐term efficient ethylene/ethane separation.^[^ ^19^ ^]^

**Figure 10:**
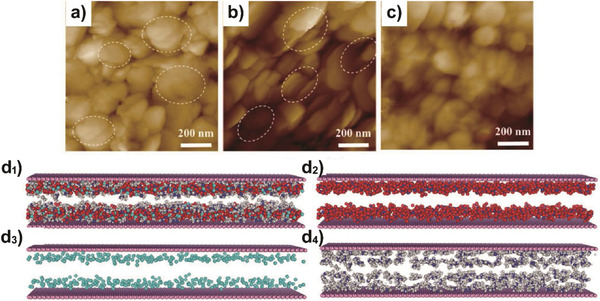
a–c) Tapping mode AFM surface images of boron nitride (BN)/nylon membrane, confirming the self‐assembly of BN nanosheets at horizontal and inclined directions. d1–d4) Snapshots of molecular simulations of reactive ionic liquids, which is composed of 1‐ethylimidazolium nitrate and silver nitrate, nanoconfined within BN nanochannels, showing the nanoaggregation of alkyl groups in ionic liquids and ordered arrangement of silver ions along the BN nanosheets; C: gray, H: white, O: red, N: blue, Ag: cyan: d1) all atoms, d2) NO_3_^−^ anion of ionic liquids and silver salt, d3) silver cation, and d4) cation of ionic liquids. Reproduced with permission.^[^ ^21^ ^]^ Copyright 2019, John Wiley and Sons.

Apart from polymers, investigations of supported membrane materials have also extended to 2D materials. Recently, Dou et al. developed a novel boron nitride (BN) membrane with a distinct nanoconfinement effect, which achieved efficient ethylene/ethane separation.^[^ ^21^ ^]^ The vacuum‐assisted self‐assembly of BN nanosheets was used to construct the BN membrane on a commercially available nylon support (BN/nylon), and spin coating was used to confine reactive ionic liquids (RIL) within the nanochannels of BN membrane through capillary force (IL‐BN/nylon). The self‐assembly of BN nanosheets at horizontal and inclined orientation was confirmed by AFM images (**Figure** **10**a–c). Small few‐layered BN nanosheets with sizes of 50–250 nm created more stable and percolating nanochannels for gas transport. The ordered nanostructure of RIL is shown by the simulation box (Figure 10d1–d4): the alkyl of cations aggregate to form non‐polar regions separated by polar regions of the aggregated NO_3_^−^ anions.

Since it has generally been found that liquid membranes suffer from a loss of membrane liquid under high operating pressure, membranes in which the carrier functions in a solid environment have been investigated. Carriers such as silver ions are generally constrained around their counter ions or chemical groups by electrostatic interaction or coordination. Thus, carriers with this type of microenvironment are referred to as semi‐mobile carriers or fixed carriers. In a solid microenvironment, the carrier has less mobility than in a liquid environment. In 1986, Kraus et al. studied water‐free facilitated transport membranes through an ion‐exchange membrane with the addition of plasticizer (glycerol or other polyhydric alcohol).^[^ ^98^ ^]^ However, the performance declined over time due to the loss of plasticizer. Subsequently, many fixed carrier membranes based on polymers, including poly(phenylene oxide) (PPO), poly(vinyl methyl ketone), poly(ethylene glycol), polyvinylpyrrolidone, and poly(2‐ethyl‐2‐oxazoline) (POZ), were investigated. Liao et al. investigated fixed site carriers in a rigid network structure by loading silver ions in hydrolyzed PIM‐1 matrix.^[^ ^99^ ^]^ Another type of solid microenvironment is a framework structure, whereby the carrier is confined within ordered framework channels. Sakai et al. studied Ag‐exchanged X‐type zeolite membrane, which was prepared from Na‐X membrane by ion exchange with AgNO_3_.^[^ ^47c^ ^]^ The introduction of Ag+ improved the propylene/propane selectivity considerably in binary mixture tests, from about 4.4 in Na‐X membranes to above 50 in fully Ag‐exchanged Na‐X membranes. Carriers in network structures have been widely studied, while the distribution and reactivity of carriers in frameworks need further research.

Luangrujiwong et al. evaluated the performance of carriers in both liquid and solid environments.^[^ ^95^ ^]^ Although the solid environment addressed mechanical stability issues, it showed that the ethylene permeability of carriers in liquid microenvironment was higher. Therefore, there is much room to improve the separation performance of fixed site carrier membranes.

#### 2.2.2 Carrier Activity

The carrier activity is determined by the transition metal counter ions and functionality in the membrane matrix. Counter ions mainly affect the activity of carriers through the lattice energy, which is the potential energy required to break apart an ionic solid and convert its component atoms into gaseous ions. Taking Ag(I) as an example, the larger the lattice energy of the salt, the stronger the electrostatic interaction between the positive and negative ions. Thus, the dissociation ability of the silver ion will become weaker and complexation with olefin will be not strong enough. Kang et al. summarized the lattice energy of representative transition metal salts.^[^ ^100^ ^]^ Counterions BF_4_^−^ and CF_3_SO_3_^−^ have the lowest lattice energy. Thus, many polymer electrolyte membranes introduce AgBF_4_ or AgCF_3_SO_3_ as carriers. Kang et al. demonstrated that AgBF_4_, which has a low lattice energy of 658 kJ mol^−1^, exhibits a higher selectivity to propylene than AgCF_3_SO_3_ (719 kJ mol^−1^) for the same polymer matrix and silver salt filling ratio.^[^ ^101^ ^]^ Sunderrajan et al. first studied olefin adsorption behavior in solid polymer electrolytes based on blends of poly(ethylene oxide) (PEO) and different silver salts.^[^ ^91^ ^]^ The order of olefin solubility in blends containing 1 mol of silver ions per mole of ethylene oxide units is: AgBF_4_ (0.49 mol/mol Ag+)>>AgCF_3_SO_3_ (0.13 mol/mol Ag^+^)>AgCF_3_CO_2_ (0.06 mol/mol Ag^+^)>AgNO_3_ (0.03 mol/mol Ag^+^), which is fully consistent with the sequence of lattice energy.

Functional groups in membrane matrices also exert a great influence on the activity of carriers. Kang et al. elucidated the role of different polymer structures in facilitated transport membranes by XPS, FT‐Raman and FT‐IR.^[^ ^101^ ^]^ The interaction intensity between silver ions and various ligands follows the order of amide > ketone > ester. The relative strength of interaction of silver ions between different polymer ligands and olefin molecules also influences the concentration of olefin carriers. The concentration is high when the former interaction is stronger than the latter and vice versa. It is well known that crown ethers can complex with numerous metal ions including silver ions. Hess et al. prepared membranes containing crown ethers and loaded Ag^+^ as carriers.^[^ ^102^ ^]^ It was found that the propylene/propane selectivity increased with increased loading of crown ether. This indicates that the crown ether weakens the electrostatic interaction of the silver salt and promotes higher carrier activity.

We can conclude that the activity of ion‐type olefin carriers is related to their interaction with counter ions, chemical groups, and olefin molecules. There are competing relationships between these three kinds of interactions. In detail, the electrostatic interaction between counter ions and carriers hinders the release of the carrier from its salt. Also, if the coordination between chemical groups and carriers is too strong, it will hinder the complexation of carrier with olefin molecules. Conversely, a weak interaction between chemical groups and carriers cannot facilitate the release of carriers from its salts. If the complexation of the olefin molecule with the carrier is too weak, it will be insufficient for chemisorption. Conversely, too strong an interaction will hinder the desorption of olefin molecules. Therefore, studying the three competitive interactions and making a reasonable balance are beneficial to improving the activity of carriers to facilitate olefin transport.

In the case of silver nanoparticles as carriers, it is necessary to increase the activity of the nanoparticles by regulating the chemical microenvironment. If only silver nanoparticles are used, even when dispersed in ILs, the selectivity is generally not high (only 14.4 for propylene/propane). Through incorporating strong electron acceptors, the surface energy level and charge distribution of the metal can be adjusted to increase the carrier activity of the nanoparticles. Kang et al. used *p*‐benzoquinone (p‐BQ) and tetracyanoquinodimethane (TCQN) as electron acceptors to increase carrier activity of Ag nanoparticles.^[^ ^103^ ^]^ Moreover, since the electron affinity of TCQN (+2.8) is stronger than that of p‐BQ (+1.86), only a small amount of TCQN (0.01 fraction of polymer weight) was found to significantly improve propylene/propane separation performance of the membrane.

In this section, we have provided an overview of the structure of carrier‐based olefin/paraffin separation membranes, including carrier types and physical/chemical microenvironments in which the carriers are located. Manipulating the physical and chemical microenvironments, selecting appropriate carriers, and regulating the membrane structure will be beneficial to improve the performance of carrier‐based separation membranes. In addition, the adsorbent prepared by Li et al., which adsorbs paraffins preferentially, may represent a promising new direction for the development of carrier‐based membranes.^[^ ^104^ ^]^

## 3 Physical and Chemical Transport Mechanisms within Membranes

In general, olefin/paraffin separations are based on the physical mechanisms in channel‐based membranes and the chemical mechanisms in carrier‐based membranes. For physical mechanisms, we focus on the solution–diffusion mechanism in membranes with network structures and the molecular sieving mechanism in membranes with framework structures. For chemical mechanisms, we focus on the facilitated transport mechanism in carrier‐based membranes.

### 3.1 Solution‐Diffusion

**Figure 11:**
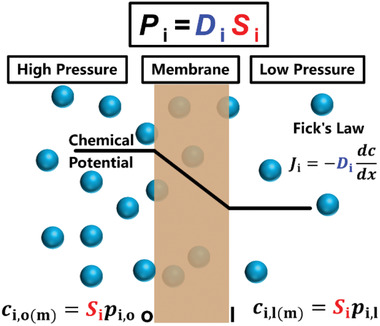
Representation of the solution–diffusion model of gas transport in dense polymer membrane, where the gradient in chemical potential is produced by the difference in partial pressure of component i across the membrane. Reproduced with permission from Zachary P. Smith.^[^ ^105^ ^]^

For membranes with network structure, the physical mass transport of the gas molecules is usually described by the solution–diffusion mechanism consisting of three steps. Gas molecules first dissolve and are adsorbed on the upstream surface of the membrane. Then, the dissolved gas molecules diffuse within the membrane and reach the downstream side under a driving force of partial pressure difference. Finally, gas molecules desorb on the downstream surface (**Figure** **11**).^[^ ^105^, ^106^ ^]^ The permeability (*P*_i_) of gas i can be described as follows:

(3) $$P_{i}=\frac{Q_{i}l}{\Delta p_{i}A}\;$$

where the *Q_i_* (cm^3^(STP) s^−1^) represents the flux of component i across the membrane; *l* (cm) represents the thickness of the membrane; *Δp*_i_ (cmHg) is the partial pressure difference of gas i through the membrane; and *A* (cm^2^) is the area of the membrane. The unit of permeability is Barrer, which is 10^−10^ cm^3^(STP)·cm·cm^−2^·s^−1^·cmHg^−1^.

Based on the solution–diffusion mechanism, the permeability of gas is the product of the solubility coefficient (*S*, cm^3^(STP)·cm^−3^·cmHg^−1^) and the diffusion coefficient (*D*, cm^−2^·s^−1^)^[^ ^107^ ^]^

(4) $$P_{i}=S_{i}\times D_{i}$$

Gas permselectivity (*α*_i/j_) is defined as the ratio of permeabilities (*P*_i_/*P*_j_), which can further be expressed as the product of solubility selectivity (thermodynamic) and diffusivity selectivity (kinetic)

(5) $$\alpha_{i/j}=\frac{P_{i}}{P_{j}}=\frac{D_{i}\times S_{i}}{D_{j}\times S_{j}}=\frac{D_{i}}{D_{j}}\times \frac{S_{i}}{S_{j}}$$

For a specific network material, the solubility depends mainly on the interaction between gas molecules and membrane materials, especially the condensability of gas molecules. The gas molecules which show higher condensability are more soluble in network materials. The condensability is related to the saturated vapor pressure of the gas. The lower the saturated vapor pressure, the more easily the gas will condense. The diffusivity mainly depends on the molecular size and shape.^[^ ^108^ ^]^ Gas molecules with smaller size diffuse faster in network structures.^[^ ^109^ ^]^

For olefin/paraffin mixtures, the solubility selectivity is close to unity in network materials and other membrane materials where only physical adsorption occurs because of the similar boiling points between respective C2 or C3 olefin and paraffin molecules. Simultaneously, the free volume in membranes with network structures cannot effectively distinguish between olefins and paraffins because of their small differences in molecular size and the random and transient properties of free volumes. Thus, diffusivity selectivity is low, which is why many membranes with network structures exhibit low separation performance.

### 3.2 Molecular Sieving

**Figure 12:**
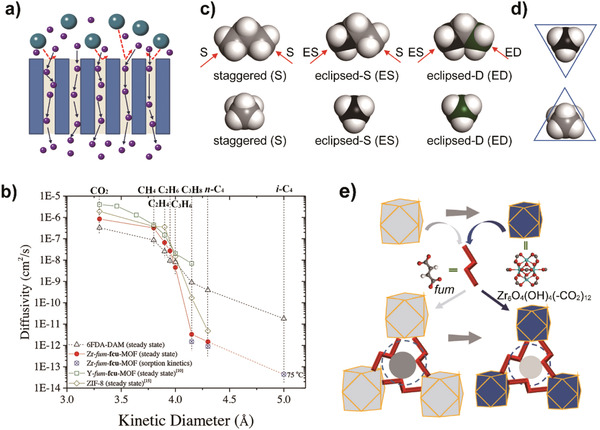
a) A schematic of molecular sieving separation of molecules of two different sizes in membrane pores. Larger‐diameter molecules are excluded by molecular sieving. b) Diffusion coefficients of eight gases (CO_2_, CH_4_, C_2_H_4_, C_2_H_6_, C_3_H_6_, C_3_H_8_, *n*‐C_4_H_10_, and *i*‐C_4_H_10_) in Zr‐fum‐fcu‐MOF and 6FDA‐DAM at 35 °C and 106 kPa. The diffusivities of C_3_H_8_, *n*‐C_4_H_10_, and *i*‐C_4_H_10_ were also estimated from their sorption kinetics of Zr‐fum‐fcu‐MOF crystals; c) Typical conformers of a C_3_H_8_ molecule in the space‐filling (CPK) model: staggered (S), eclipsed‐S (ES), and eclipsed‐D (ED) representing conformers with one end in eclipsed conformation and both ends in eclipsed conformation, respectively. The carbons in the rotated ‒CH_3_ are shown in different colors (black and green) for easier observation. d) Hypothesized triangular aperture that can distinguish “eclipsed” conformer (up) from “staggered” conformer (down) with the solid line triangle in the front plane. e) Schematic representation of the pore‐aperture size reduction of fum‐fcu‐MOF induced by reduced metal cluster size from Y‐fum‐fcu‐MOF (left) to Zr‐fum‐fcu‐MOF (right). Reproduced with permission.^[^ ^81^ ^]^ Copyright 2019, John Wiley and Sons.

For a single pore, when the aperture is between the sizes of two gas molecules, only the smaller molecules can diffuse through the pore and the larger molecules will be rejected. This phenomenon is referred to as molecular sieving. If the membrane material is filled with such pores of uniform size and orientation, there will be a pronounced molecular sieving effect, allowing penetration of only the smaller molecules (**Figure** **12**a). However, in actual situations, due to the presence of structural defects, orientation, and other imperfections, ideal molecular sieving rarely occurs and the membrane may exhibit a sudden drop in gas permeance with an increase of gas kinetic diameter (Figure 12b).^[^ ^61^ ^]^

The molecular sieving effect can be regarded as a diffusion‐dominated penetration process. Fick and Jacobs considered diffusion in a variable‐section pipeline as 1D diffusion, which means the entropy barrier of diffusion is determined by the cross‐sectional area or width of the pipeline.^[^ ^110^ ^]^ In the case of small curvature of variable section, the theory is quite reliable.^[^ ^111^ ^]^ On the other hand, diffusion is an activated process in both network structures and framework structures, so the diffusion coefficient (*D*_A_) can be expressed as an Arrhenius relationship^[^ ^112^ ^]^

(6) $$D_{A}=D_{0A}\exp\left(\frac{-E_{\mathrm{DA}}}{RT}\right)$$

where *R* is the molar gas constant, *T* is the temperature, and *D*_0A_ and *E*_DA_ are the pre‐exponential factor and activation energy for the diffusion of penetrant A, respectively. Furthermore, according to the theory of transition state theory, the pre‐exponential factor can be represented as follows:

(7) $$D_{0A}=e\lambda^{2}\frac{kT}{h}\exp\left(\frac{S_{D}}{R}\right)$$

where *λ* is the average diffusive jump length, *S*_D_ is the activation entropy of diffusion, *k* is Boltzmann's constant, and *h* is Planck's constant. For gas pairs with similar kinetic diameters that are able to access all of the micropores, *λ* can be considered equal for both gases. However, for gas molecules whose size is much larger than the aperture, the diffusional jump length is usually 0 and the diffusion coefficient is 0. Combined with the above two equations, the diffusion selectivity is obtained as follows:

(8) $$\begin{array}{ccc} \frac{D_{A}}{D_{B}} & = & \left(\frac{{\lambda_{A}}^{2}}{{\lambda_{B}}^{2}}\right)\frac{D_{0A}}{D_{0S}}\exp\left(-\frac{E_{\mathrm{DA}}-E_{\mathrm{DB}}}{RT}\right)\\ & = & \left(\frac{{\lambda_{A}}^{2}}{{\lambda_{B}}^{2}}\right)\underset{\mathrm{Entropic}\;\mathrm{selectivity}}{\underset{︸}{\exp\left(\frac{S_{\mathrm{DA}}-S_{\mathrm{DB}}}{R}\right)}}\cdot \underset{\mathrm{Enthalpic}\;\mathrm{selectivity}}{\underset{︸}{\exp\left(-\frac{E_{\mathrm{DA}}-E_{\mathrm{DB}}}{RT}\right)}}\\ & = & \left(\frac{{\lambda_{A}}^{2}}{{\lambda_{B}}^{2}}\right)\exp\left(\frac{\Delta S_{\mathrm{DA},B}}{R}\right)\cdot \exp\left(-\frac{\Delta E_{\mathrm{DA},B}}{RT}\right) \end{array}$$

In other words, the diffusion process is determined by the diffusion activation energy and the diffusion activation entropy. The diffusion selectivity is further divided into enthalpic selectivity and entropic selectivity.^[^ ^113^ ^]^ Activation energy refers to the energy required for the molecule to move from the ground state to the diffused active state, including the energy required for the molecule to desorb from the sorption site, *E*_desorp_, and the energy needed to overcome the repulsion of the micropores, *E*_repulsion_,^[^ ^114^ ^]^

(9) $$E_{D}=E_{\mathrm{desorp}}+E_{\mathrm{repulsion}}$$

For materials that rely on increasing sorption to improve selectivity, this will inevitably lead to greater desorption energy of the target molecule, resulting in an increase in activation energy. On the other hand, *E*_repulsion_ is related to the size of the gas molecule. The smaller the size of molecules, the smaller the energy required and the lower the activation energy. If the size of the gas molecules is larger than the size of the micropores, then the jump length shrinks considerably and the repulsion force of the micropores increases to be nearly infinite. In this case, the activation energy is very large and diffusion of the gas molecules is difficult.

Activation entropy refers to the change of standard entropy of gas molecules from the ground state to the diffusion activation state, which is related to the microscopic state of the gas molecules. Based on transition state theory, the diffusion of gas molecules through a medium can be described by the following equation:

(10) $$D=\lambda^{2}\frac{kT}{h}\frac{F^{\neq }}{F}\exp\left(\frac{-E_{D}}{RT}\right)$$

where *F* ^≠^ and *F* are the partition functions of gas molecules in the ground state and when they are passing through the micropores, respectively. In the microscopic state, molecules have three modes of motion: translational, rotational, and vibrational. The partition function can also be regarded as the product of these three functions.

(11) $$F=F_{\mathrm{trans}}\times F_{\mathrm{rot}}\times F_{\mathrm{vib}}$$

Normally, the vibrations of molecules are invariable, while the translational and rotational states are related to the shape of the gas molecules and micropores. Considering a suitable slit pore geometry, the translational and rotational degrees of freedom of ethylene molecules are 2, while the translational and rotational degrees of freedom of ethane molecules are 1 and 0, respectively. Moreover, the partition function of ethylene molecules is larger than that of ethane molecules, and the activation entropy of ethylene molecules is larger, which leads to entropy selectivity and enhances the effect of molecular sieving.

Liu et al. investigated the molecular sieving effect of triangular pores on propylene/propane gas pairs by preparing Zr‐fum‐fcu‐MOF.^[^ ^81^ ^]^ Molecular simulations showed that propylene could transit smoothly, whereas propane could only pass in a double eclipsed conformation (Figure 12c). It requires a high rotational barrier for propylene to reach the double overlap conformation (Figure 12d,e). Therefore, the barrier of propane through the triangular hole increases, which endows the triangular hole with a better sieving ability (Figure 12e). This confirms the effect of micropore shape on molecular sieving effect.

From the above, we can draw some brief conclusions. First of all, in order to achieve a better molecular sieving effect, rigid pores are more effective than flexible ones. Second, not only is the size of micropores important, but also the conformation or shape of the micropores and gas pairs. For the selection of molecular size, the L‐J diameter takes into account the influence of molecular shape, so it is better to choose the L‐J diameter as a reference rather than van der Waals diameter in olefin/paraffin separations.

### 3.3 Facilitated Transport

**Figure 13:**
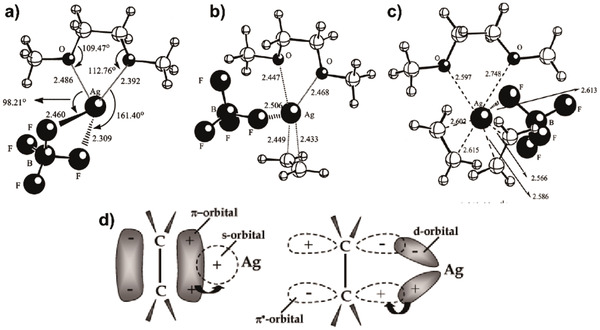
Optimized structures of a) the PEO/AgBF_4_ complex, b) the PEO/AgBF_4_ complex coordinated by one ethylene, and c) the PEO/AgBF_4_ complex coordinated by two ethylene molecules (transition state). Reproduced with permission.^[^ ^115^ ^]^ Copyright 2001, American Chemical Society. d) Schematic representation of Ag^+^‐propylene complexation. Reproduced under the terms of the Creative Commons Attribution 4.0 International License.^[^ ^116^ ^]^ Copyright 2006, the Authors, Published by Brazilian Society of Chemical Engineering.

In order to analyze the mechanism of facilitated transport, we should first understand the process of the adsorption and desorption processes between carriers and molecules. For AgBF_4_ and ethylene, the reaction mechanism can be elucidated by calculating the theoretical structure and the electronic energies (**Figure** **13**a–c).^[^ ^115^ ^]^ When an ethylene molecule is introduced into the BF_4_‐Ag‐PEO complex (a), a new complex (b) is formed by replacing one site of the anionic ligand with an ethylene molecule. The olefin molecule supplies *π* electrons from the occupied 2p orbital to the empty s orbital of the silver ion to form a *σ* bond; the silver ion feeds back the electron of the d orbital to the π*‐2p anti‐bond orbital of the olefin molecule to form a *π* bond, as shown in Figure 13d. When another ethylene molecule approaches the complex (b), the ethylene molecule that has been bound to the silver ion will be replaced by a new ethylene molecule, which is activated by a normal push–pull S_N_2 transition state (c).

(12) $$\mathrm{olefin}+\mathrm{carrier}\underset{k_{r1}}{\overset{k_{f1}}{⇄}}\mathrm{complex}$$

(13) $$\mathrm{olefi}n_{1}+\mathrm{comple}x_{1}\underset{k_{2}}{\overset{k_{2}}{⇄}}\mathrm{comple}x_{2}+\mathrm{olefi}n_{2}$$

that is,

(14) $$C_{2}H_{4}+\mathrm{Ag}\left(I\right)⇄\left[\mathrm{Ag}\left(I\right)\left(C_{2}H_{4}\right)\right]$$

(15) $$\begin{array}{ccc} C_{2}H_{4}+\left[\mathrm{Ag}\left(I\right)\left(C_{2}H_{4}\right)\right] & ⇄ & \left[\mathrm{Ag}\left(I\right){\left(C_{2}H_{4}\right)}_{2}\right]\\ & ⇄ & \left[\mathrm{Ag}\left(I\right)\left(C_{2}H_{4}\right)\right]+C_{2}H_{4} \end{array}$$

As mentioned above, carriers in different matrices have different mobility capabilities, and the facilitated transport mechanisms are also different. Both systems include gas molecules, olefin‐carrier complexes, and uncoordinated carriers. The latter two are free to move in a liquid environment. Thus, a chemical potential difference exists between the upstream and downstream sides of the membrane with spontaneous diffusion. These two kinds of carriers are distributed in accordance with the chemical potential difference. The carrier in the liquid environment mainly undergoes the reaction in Equation (12) and physical diffusion. The carrier in the solid environment mainly undergoes chemical diffusion of Equation (13) with a reaction of Equation (12) occurring on both sides of the membrane.

The flux of carrier‐based membrane in a liquid environment, *J*_A_, can be illustrated as the sum of Fickian diffusion and carrier‐mediated diffusion^[^ ^117^ ^]^

(16) $$J_{A}=\frac{D_{A}}{l}\Delta C_{A}+\frac{D_{\mathrm{AC}}}{l}\Delta C_{\mathrm{AC}}$$

In a typical reaction‐controlled facilitated transport process, the membrane performance is highly dependent on the reaction of Equation (12) because the first step is not completely reversible and is relatively slow. First, the *K* value relates to the reactant directly. The *π* complexation reaction can be regarded as a kind of soft and hard acid–base reaction. Olefins provide electrons and are considered to be soft bases. The soft acid that accepts electrons facilitates complexation with olefins. For soft acid and soft base reactions, the energy difference between the HOMO orbital of the base and the LUMO orbital of the acid is small, so electrons can migrate easily. The electron‐withdrawing ability of a carrier, that is, electronegativity, determines the strength of its complexation with olefin molecules. Transition metal ions having an electronegativity between 1.6 and 2.3 are suitable as carriers for promoting the transport of olefin molecules.^[^ ^100^ ^]^ That is why Ag(I), Cu(I), etc., have the best performance among the transition metals. On the other hand, for the interaction between propylene and silver ions, Ortiz et al. established a model through adsorption experiments in 2008, and proved that the complexation enthalpy of propylene–silver complexes with a 1:1 stoichiometry was −11.0 kJ mol^−1^.^[^ ^118^ ^]^ Thus, the equilibrium constant of propylene/Ag^+^ complexation decreases as the temperature increases, which is consistent with the olefin flux decreasing as temperature increases observed in other studies.^[^ ^119^ ^]^

For facilitated transport with carriers in a solid microenvironment, the situation is quite different from that in the liquid environment because of chemical diffusion. There are several theories about the mass transfer mechanism of fixed carrier membranes. The dual adsorption model, which was originally used to explain the adsorption behavior of gases in glassy polymers, indicated the promotion of transport in the early stages.^[^ ^120^ ^]^ Later, Noble optimized the diffusion model to analyze the mechanism by introducing effective diffusion coefficients between the fixed site carriers.^[^ ^121^ ^]^ In 1989, Cussler et al. developed a concept of “limited mobility of chained carriers.”^[^ ^122^ ^]^ The theory holds that the carriers are not completely immobile but have a certain ability to move. The membrane is divided into a number of layers with thickness of *l* and each layer comprises a single layer of carrier. Each carrier can move a distance *l*_0_ near the equilibrium position within a layer, but no carrier can permanently move from one layer to another. The kinetic ability of the carrier and the state of dispersion of the carrier in the membrane together determine the mass transfer rate of the gas molecules within the membrane.

As shown in the above model, the apparent mass transport process may not reflect the diffusion process but the chemical kinetics. Furthermore, because the mobility of carriers in a solid microenvironment is restricted, the concentration of carriers will have a threshold to exhibit a pronounced facilitated transport effect. This theory embodies the difference and connection between facilitated transport mechanisms of carriers in these two different microenvironments. The stratified approach may also guide the transport mechanism research in 2D materials or framework materials.

## 4 Membrane Application in Olefin/Paraffin Separations

### 4.1 Ethylene/Ethane Separation

As mentioned previously, ethylene and ethane molecules possess very similar physical properties. The differences in boiling point and molecular size (Lennard–Jones diameter) are only 15 K and 0.19 Å, respectively. Therefore, it is difficult to separate ethylene/ethane mixture with only the solution‐diffusion mechanism, which primarily depends on the condensability and molecular size. Thus, differences in the chemical bonding ability could play an important role in enabling the efficient separation of ethylene and ethane, as elaborated in the section of facilitated transport mechanism. Furthermore, ethylene molecules have a planar configuration while ethane molecules have a spatial configuration. The configuration differences may also contribute to separating ethylene from ethane efficiently.

**Figure 14:**
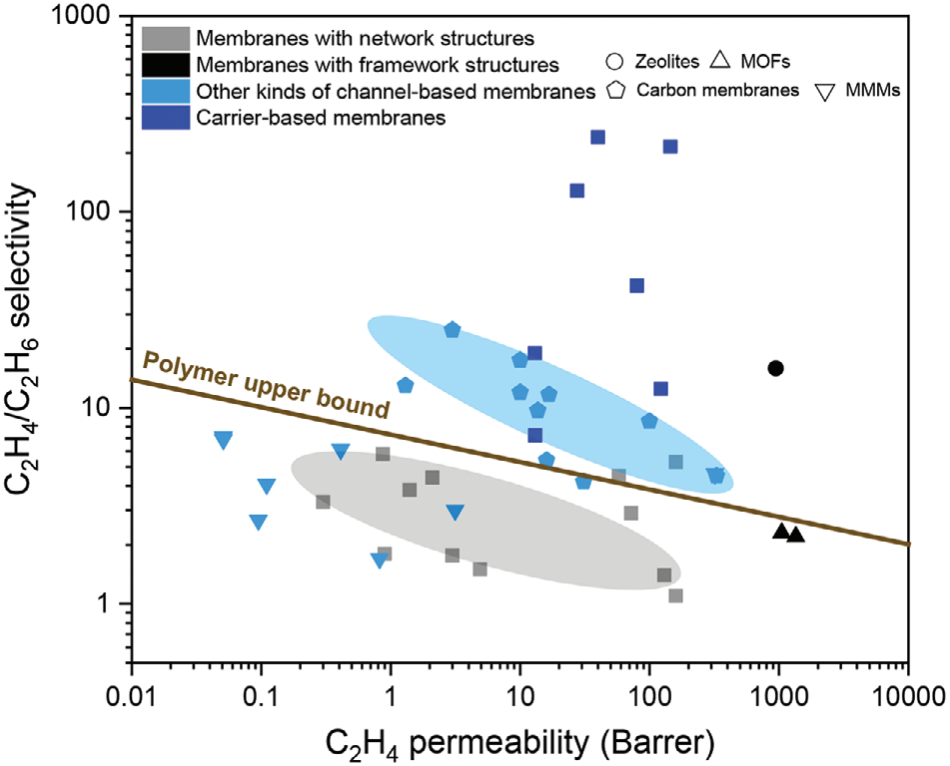
Ethylene/ethane separation performance of channel‐based and carrier‐based membranes. The polymer upper bound is represented as a bold solid line according to ref. [123]. The grey and light blue oval areas highlight the separation performance of membranes with network structures and other kinds of channel‐based membranes, respectively. Membranes with framework structures are sub‐divided into zeolite and MOF membranes, represented by different shapes. Other kinds of channel‐based membranes are further sub‐divided into carbon membranes and MMMs, represented by different shapes. Here, membranes with network structures specifically refer to polymeric membranes. Carrier‐based membranes specifically refer to facilitated transport membranes. These two classes of membranes are represented by squares, but distinguished by different colors.

So far, most of the channel‐based membranes used for ethylene/ethane separation have been membranes with network structures. Thus, membrane structures and separation performance have been widely investigated on polymeric membranes. Usually, polymeric membranes separate ethylene/ethane by the solution‐diffusion mechanism. Because the solubility ratio of ethylene to ethane is almost unity, it is the differences in the diffusion ratio through the polymer matrix that determines the overall selectivity. Thus, the trade‐off effect between permeability and selectivity dominates the separation performance of polymeric membranes, as shown in **Figure** **14** and Table S1, Supporting Information. For conventional polyimide membranes, the dense membrane structure and chain rigidity confer moderate ethylene/ethane selectivity, but with very low ethylene permeability. Staudt‐Bickel et al. constructed three different structures of 6FDA‐based polyimide membranes and all exhibited ethylene permeability of lower than 3 Barrer with ideal ethylene/ethane selectivity of about 4.^[^ ^26e^ ^]^ Another 6FDA‐TrMPD membrane prepared by Tanaka et al. exhibited higher ethylene permeability of 58 Barrer, but with predictably lower ethylene/ethane selectivity of 2.9.^[^ ^26b^ ^]^ Further, Rungta et al. summarized the ethylene/ethane separation performance of pure gases using various polymeric membranes, which were carried out under standard conditions at 35–50 °C and 1–2 atm feed pressure with downstream under vacuum.^[^ ^123^ ^]^ The trade‐off effect was quantitatively analyzed and the upper bound of ethylene/ethane separation was also predicted to be similar to other important gas pairs, based on the theoretical basis for the upper bounds, put forward by Freeman.^[^ ^124^ ^]^ However, the overall separation performance of polymeric membranes is not sufficient to satisfy practical applications, especially since the selectivity of most polymeric membranes is lower than 5. This is mainly because free volume elements, which are the transport channels in polymeric membranes, are transient and continuously changing without specific shapes. Besides, the difference of molecular size and the interaction with polymer matrix between ethylene and ethane is too small. Therefore, the enhancement of molecular sieving effect and/or sorption or facilitated transport capability toward target molecules is essential to improve separation performance.

In order to enlarge the diffusional difference between ethylene and ethane, various carbon membranes with molecular sieving effect (or carbon molecular sieve membranes, CMS membranes) have been investigated. Generally, the optimized ethylene/ethane separation performance of CMS membranes surpasses the upper bound of polymeric membranes, and especially the selectivity is substantially improved, even up to 25, owing to the enhancement of diffusivity selectivity.^[^ ^125^ ^]^ Carbon membranes possess rigid ultramicropores which enable efficient separation of similarly‐sized gas pairs (such as ethylene and ethane). This is because the slight differences in molecular size result in considerable differences in the activation energy required to make a diffusive jump, referred to as “enthalpic selectivity”. In addition, the rigid slit‐like ultramicropores restrict the degrees of freedom of rotation of molecules and allow preferential transport of ethylene with planar configuration and slimmer size, referred to as “entropic selectivity.” Therefore, compared with polymeric membranes, CMS membranes possess high entropic selectivity and exhibit high ethylene/ethane selectivity. Rungta et al. prepared Matrimid‐derived CMS membranes with ethylene permeability of about 14 Barrer and high ethylene/ethane selectivity of 12.^[^ ^85c^ ^]^ The optimized pyrolysis temperature of 675 °C ensured both a high ethylene/ethane selectivity and moderate ethylene permeability. A further modified pyrolysis protocol with a faster heating rate was demonstrated to contribute to increasing permeability without sacrificing selectivity. Therefore, comprehensive optimization of pyrolysis parameters is needed to achieve both high permeability and high selectivity. Microporous organic polymer membranes also present new opportunities for polymeric membranes, since they enlarge the field of available precursors for CMS membranes, especially PIMs and polyimides with intrinsic microporosity. Although PIM‐1 membrane exhibits outstanding separation performance toward some important gas pairs, such as O_2_/N_2_ and CO_2_/CH_4_, its olefin/paraffin separation performance is poor (ethylene/ethane selectivity of about 1.4). This is mainly because of the relatively large micropore size (about 7 Å) with a broad pore size distribution. Thermal pre‐treatment has been proposed to reduce the pore size and narrow the pore size distribution, which are necessary to improve molecular sieving. Salinas et al. prepared PIM‐1‐derived CMS membranes and obtained an ethylene/ethane ideal selectivity of 13, but with a low ethylene permeability of 1.3 Barrer.^[^ ^86e^ ^]^ CMS membranes derived from a polyimide with intrinsic microporosity (PIM‐6FDA‐OH) were fabricated by the same group by pyrolysis at 800 °C, which had an ideal ethylene/ethane selectivity of 17.5 with ethylene permeability of 10 Barrer.^[^ ^86f^ ^]^ The high selectivity was mainly the result of reduced pore size and narrow pore size distribution from pore collapse at the higher pyrolysis temperature.

Carrier‐based membranes have the capability to separate olefin/paraffin gas pairs effectively based on the facilitated transport mechanism, that is, the stronger interaction between olefins and metal ions (such as Ag^+^ and Cu^+^) in the membrane matrix through *π*‐coordination. Liquid membranes, especially SLMs, are one of the most widely researched carrier‐based membranes. Liquid membranes usually exhibit higher separation performance than solid membranes because of faster transport in the liquid phase and the high activity of facilitated transport carriers. Dou et al. introduced Ag^+^ into protonic ILs and achieved ethylene permeability of 244 Barrer with a high ethylene/ethane selectivity of 57.^[^ ^126^ ^]^ The good compatibility between carriers and protonic ILs, high solubility of carriers, and favorable polar domains for the transport of carrier–ethylene complex contributed to the outstanding separation performance. Apart from introducing metal salts into ILs, integrating metal ions as the cation moiety of ILs is an ingenious strategy to achieve intrinsically high carrier concentration.

Polymer electrolyte composite membranes are another type of carrier‐based membranes, whereby the metal salts are dispersed into rubbery heteroatom‐containing polymer matrices and dissociated into cations and anions.^[^ ^127^ ^]^ The lone pair electrons in the polymer chains help to fix metal ions through coordination bonding. Using ion exchange, a silver‐containing sulfonated PPO membrane was reported in 1980 by Leblanc et al., which had an ethylene/ethane selectivity of 288.^[^ ^128^ ^]^ In 2001, Pinnau et al. fabricated a PEO membrane loaded with AgBF_4_ and found that both the ethylene permeability and ethylene/ethane selectivity increased dramatically when the AgBF_4_ concentration exceeded 50 wt%.^[^ ^127^ ^]^ This result demonstrated that the carriers in polymer electrolyte membranes are not freely mobile and that the hopping mechanism dominates the facilitated transport. Therefore, a threshold carrier concentration is needed for effective facilitated transport. It is also necessary to pay attention to the compatibility between metal salts and polymer matrix when the concentration of metal salts is high.

2D nanosheets have the potential to provide ordered and confined structures and a unique platform to achieve facilitated transport through metal ion‐containing solution impregnation. BN membrane modified with Ag^+^‐containing reactive ILs was prepared by Dou et al. and investigated for gas separation for the first time.^[^ ^21^ ^]^ An ordered alignment of cations and anions of the ILs was formed due to the noncovalent interaction between BN nanosheets and ILs. The nanoconfinement effect owing to the alignment of ionic liquids constructed a continuous distribution of Ag^+^ and a favorable microenvironment for Ag^+^. Thus, the Ag^+^ possessed high activity and the modified BN membrane exhibited superior separation performance with ethylene permeance of 138 GPU and ethylene/ethane selectivity of 128. Further, graphene oxide (GO) membranes, which are the most widely researched 2D membranes, with facilitated transport mechanism were explored.^[^ ^129^ ^]^ In detail, Ag^+^ was introduced as facilitated transport carriers and ILs were impregnated into the GO galleries, generating slit‐like pores in the plane of GO nanosheets to enhance the molecular sieving effect and stabilizing carrier activity. Thus, modified GO membranes with dual transport mechanisms exhibited super‐high ethylene/ethane selectivity of 215 with ethylene permeance of 72.5 GPU, surpassing most of the state‐of‐the‐art membrane towards ethylene/ethane separation.

### 4.2 Propylene/Propane Separation

Similar to ethylene/ethane separation, propylene/propane separation is one of the challenging separation systems. The differences of molecular size (Lennard–Jones diameter) and boiling point are 0.38 Å and 5.6 K, respectively. Thus, it is easier to achieve better permselectivity for propylene/propane compared with ethylene/ethane because of the larger difference in molecular size. Because the molecular sizes of propylene and propane are larger than that of ethylene and ethane, the permeability of C3 molecules (propylene and propane) is usually smaller compared to C2 molecules (ethylene and ethane).

**Figure 15:**
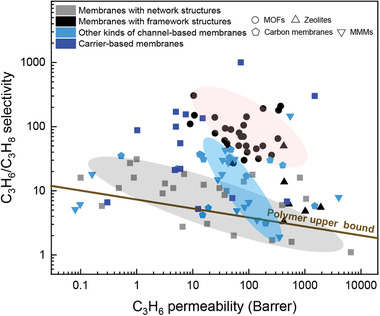
Propylene/propane separation performance of channel‐based and carrier‐based membranes. The polymer upper bound is represented as a bold solid line according to ref. [130]. The grey, light pink, and light blue oval areas highlight the separation performance of membranes with network structures, membranes with framework structures, and other kinds of channel‐based membranes, respectively. Membranes with framework structures are sub‐divided into zeolite and MOF membranes, represented by different shapes. Other kinds of channel‐based membranes are further sub‐divided into carbon membranes and MMMs, represented by different shapes. Here, membranes with network structures specifically refer to polymeric membranes, while carrier‐based membranes specifically refer to facilitated transport membranes. These two classes of membranes are represented by squares, but distinguished by different colors.

Channel‐based membranes and carrier‐based membranes, as well as carbon membranes have been widely investigated for propylene/propane separation and have exhibited competitive separation performance, as shown in **Figure** **15** and Table S2, Supporting Information. For a detailed elaboration about carbon membranes and carrier‐based membranes, refer to the contents in Section 4.1. Here, we highlight membranes with network structures and framework structures.

As mentioned before, the ethylene/ethane selectivity of polymeric membranes is usually less than 5. However, the propylene/propane selectivity of some glassy polymer membranes exceeds 30.^[^ ^26e^ ^]^ The solubility selectivity of propylene/propane is close to unity and the relatively high selectivity is due to larger diffusivity differences. Polyimide membranes are promising in propylene/propane separation because of the wide structural tunability and good thermal stability. Yoshino et al. reported that a 6FDA/BPDA(1/1)‐DDBT copolyimide membrane exhibited a high selectivity of 31, even though the propylene permeability was low (1 Barrer).^[^ ^14^ ^]^ Okamoto et al. reported a 6FDA‐TrMPD membrane having a lower selectivity of 11, but with higher propylene permeability of 30 Barrer.^[^ ^26c^ ^]^ This illustrates the trade‐off effect between propylene permeability and propylene/propane selectivity in polyimides.^[^ ^130^ ^]^ A common finding to note is that the permeability of polyimide membranes is generally low. Higher *FFV* membranes such as PIMs may provide new opportunities to improve both the permeability and selectivity. Swaidan et al. reported in 2015 intrinsically microporous polyimide (PIM‐6FDA‐OH) membranes with high propylene/propane selectivity of 30 and relatively low permeability of 3.5 Barrer after thermal annealing at 250 °C.^[^ ^131^ ^]^ Then, Liao et al. prepared metal ion‐modified PIM‐1 membranes through coordination between metal ions (Zn^2+^, Mg^2+^, and Ag^+^) and carboxylated PIM‐1 derived by hydrolysis.^[^ ^99^ ^]^ The comparative pristine PIM‐1 membrane showed high propylene permeability of more than 2000 Barrer with propylene/propane selectivity of only about 3. After hydrolysis to convert nitrile groups to carboxylate, the selectivity increased to 9, with significant compromise of permeability (421 Barrer), because of stronger interchain interactions and reduced pore size and *FFV*. After metal ion coordination, enhanced permeability and selectivity were achieved simultaneously. Especially, the performance of Mg‐modified PIM‐1 membrane surpassed the proposed polymeric upper bound, with a propylene permeability of 568 Barrer and propylene/propane selectivity of 15.

Membranes with framework structures, particularly those composed of ZIF‐8, have attracted the most attention.^[^ ^132^ ^]^ In 2011, Pan et al. first reported that ZIF‐8 membrane could sharply separate ethylene/propylene and ethane/propane gas pairs.^[^ ^133^ ^]^ In the same year, Bux et al. explored ethylene/ethane separation with ZIF‐8 membrane, which showed a selectivity of only 2.2.^[^ ^17^ ^]^ In 2012, Zhang et al. demonstrated the molecular sieving effect of ZIF‐8 crystals toward the propylene/propane gas pair.^[^ ^61^ ^]^ This laid the foundation for ensuing intensive research in ZIF‐8 membranes for propylene/propane separation. In 2012, Pan et al. reported efficient propylene/propane separation using a ZIF‐8 membrane, for the first time.^[^ ^134^ ^]^ By applying secondary growth, the membrane was highly selective over a wide feed composition. An optimized performance of propylene permeance of 61.8 GPU and propylene/propane selectivity of 45 was realized. Since then, various attempts have been made to improve ZIF‐8 membranes to meet the requirements in industrial application, including separation performance, mechanical stability, and scalable fabrication.

The theoretical diffusivity selectivity of ZIF‐8 membrane is about 125 and various measurements have been taken to approach or even surpass this benchmark.^[^ ^135^ ^]^ In 2015, Pan et al. optimized the activation process of ZIF‐8 membrane by reducing the evaporation rate of solvent and achieved a separation factor of near 90.^[^ ^136^ ^]^ In 2016, Eum et al. fabricated defect‐free ZIF‐8 membranes using the IMMP method and the propylene/propane selectivity reached as high as 180 under an upstream pressure of 1 bar, which is almost the theoretical permselectivity of ZIF‐8 membrane.^[^ ^137^ ^]^ Also, the membrane maintained a high selectivity of 90 under high feed gas pressure (9.5 bar). So far, the highest propylene/propane selectivity of ZIF‐8 membranes was reported by Zhou et al. in 2018.^[^ ^71^ ^]^ The membrane was prepared by a fast electrochemical process which endowed the membrane with suppressed ligand movement and a stiff framework structure. Thus, an ultrahigh selectivity of 304.8 was obtained. In addition, a high selectivity could be achieved through manipulation of the grain boundary structure. Kwon et al. designed a three‐tier architecture (ZIF‐8@ZIF‐67@ZIF‐8).^[^ ^64^ ^]^ The tertiary growth of ZIF‐8 could heal potential defects occurring in the ZIF‐67 surface layer and at the grain boundary. The multiple hetero‐epitaxial growth enhanced the selectivity up to 200.

Even though membrane selectivity has been greatly improved, from several tens to more than 100 and even about 300, membrane permeance is still limited with the majority of membranes in the range of 30–60 GPU. One reason is that the thickness of most ZIF‐8 membranes is several micrometers, even up to 80 µm. Therefore, current research is focused on fabricating ultrathin MOF membranes to reduce the transport resistance. Generally, minimal thickness requires very low amounts or concentrations of reactants. Decreasing the reactant concentration to one‐tenth of those originally used led to an ultrathin ZIF‐8 membrane of 620 nm.^[^ ^70^ ^]^ Deposition‐based processes may also provide more thickness controllability than conventional solution‐only fabrication processes because the quantity of converted reactants can be more precisely controlled. Sub‐micrometer membranes were prepared by sol–vapor deposition (as low as 17 nm),^[^ ^73^ ^]^ electrochemical deposition (about 200 nm), and ALD (about 200 nm).^[^ ^71^, ^74^ ^]^ In 2017, Li et al. fabricated ultrathin ZIF‐8 membrane with thickness only about 87 nm through the GVD process.^[^ ^73^ ^]^ The thin selective layer contributed to an ultrahigh propylene permeance of 840 GPU, while retaining a good propylene/propane selectivity of about 70. They also explored the fabrication of ZIF‐8 hollow fiber modules applying the GVD technique. The performance of modules was comparable to that of small scale membranes, demonstrating the feasibility of scaling‐up ZIF‐8 membranes. For secondary growth, reducing the time of growth will benefit the decrease of membrane thickness, as demonstrated in the research of He et al.^[^ ^63^ ^]^ Besides, partially converting ZIF‐8 crystals to isomorphous analogues with larger effective aperture size is another approach for reducing effective membrane thickness. A controlled linker exchange from 2‐methyimidazole to 2‐imidazolecarboxaldehyde was conducted and partial ZIF‐8 crystals were converted to ZIF‐90 (pore size of about 5 Å) through post‐synthetic modification.^[^ ^138^ ^]^ The C_3_H_6_ permeance was greatly improved from 63 GPU to 234 GPU without significantly sacrificing selectivity.

### 4.3 Membrane Stability

Apart from the permeance and gas pair selectivity, membrane stability is also an important factor in practical application. For olefin/paraffin gas pair system, the stability herein mainly refers to the carrier stability, plasticization effect, and aging effect.

#### 4.3.1 Carrier Stability

Even though carrier‐based membranes possess outstanding separation performance, especially compared with commercial polymeric membranes, the instability of carriers (including the reduction of Ag^+^, disproportionation of Cu^+^, and poisoning by sulfides) remains the major challenges which limit the long‐term operation and practical applications. So far, various strategies have been proposed to mitigate or overcome the instability of Ag^+^ or Cu^+^.

First, replacing metal ions with corresponding metallic nanoparticles would circumvent the valence change of carriers. It is essential to induce the surface polarity of metallic nanoparticles by electron acceptors to generate activated metal ions.^[^ ^139^ ^]^ Kang et al. observed that POZ/AgBF_4_ membranes still maintained propylene/propane selectivity of 31, despite some of the Ag^+^ being transformed to Ag nanoparticles of about 27 nm.^[^ ^140^ ^]^ Further, they fabricated Ag nanoparticle‐based facilitated transport membrane polarized by *p*‐benzoquinone.^[^ ^103a^ ^]^ Because of the enhanced carrier stability, the membrane separation performance was maintained almost unchanged for 105 h.

Second, it would be beneficial to stabilize carriers by strengthening interactions between metal ions and membrane matrix (e.g., polymer chains or IL). Kang et al. found that poly(ethylene phthalate) (PEP) membrane containing AgBF_4_ had stable propylene/propane separation performance up to 150 h.^[^ ^140^ ^]^ Comparing the interaction between carbonyl groups and silver ions in PEP and other carbonyl‐containing polymers, they demonstrated that PEP exhibits a stronger interaction with Ag^+^ and deduced that a stable coordination between phthalic groups and Ag^+^ was formed. Therefore, Ag^+^ maintained a stable oxidation state and exhibited good long‐term stability. Also, Dou et al. prepared various carrier‐based membranes with IL–metal ion systems, including SLMs and 2D membranes.^[^ ^19^, ^21^, ^126^, ^129^ ^]^ These membranes exhibited steady ethylene/ethane separation performance during long‐term operation (up to 30 days). The more stable performance is mainly because favorable and protective microenvironments are constructed through IL, and specific interactions between IL and carriers (such as hydrogen bonding, electrostatic interaction, or coordination interaction) tend to stabilize the Ag^+^.

Third, some additives help to stabilize metal ions. Kang et al. added Al(NO_3_)_3_ to POZ membranes containing AgBF_4_ carriers, and the membrane performance remained steady for 14 days.^[^ ^141^ ^]^ The same strategy was extended to other polymers, including PEO and poly(vinyl alcohol).^[^ ^142^ ^]^ The stabilizing mechanism is explained as stronger interactions between BF_4_^−^ and Al^3+^, which promote the coordination between Ag^+^ and NO_3_^−^ and suppress the reduction of Ag^+^. Jose et al. proposed that phthalates help to improve the long‐term stability, which depends on the strong coordination between Ag^+^ and carbonyl groups.^[^ ^143^ ^]^

Apart from strategies to inhibit deactivation of carriers, Merkel et al. proposed a regeneration method toward reduced silver carriers.^[^ ^144^ ^]^ H_2_O_2_ and HBF_4_ were used to restore the facilitated transport capability by oxidizing reduced silver carriers. Here, it is important to note that Ag and other metal carrier activity cannot be regenerated after deactivation due to poisoning by sulfides. Thus, the elimination of sulfide species is needed to ensure facilitated transport capability of carriers. Besides, new carrier chemistry and microenvironment manipulations for carriers are needed to promote the development of facilitated transport membranes. Ethylene is one of a class of phytohormones, which impacts virtually all phases of plant growth. It has been presumed that ethylene coordinates directly to the copper cofactor in ethylene receptors, such as ETR1. The hydrophobic and reducing microenvironment combined with the electronic structures of coordinated amino acid residues contribute to the high stability and activity of Cu(I) in ETR1. This provides some possible design directions on improving carrier stabilization in facilitated transport membranes.

#### 4.3.2 Plasticization Effect

The plasticization effect is a concern, especially for polymeric membranes. Plasticization occurs when gas molecules exhibit a relatively strong interaction with polymer chains, resulting in a large number of molecules being absorbed onto the polymer matrix. This weakens the interchain interactions and the polymer chains are “swollen” or dilated by the gas. Plasticization is manifested by decreasing gas permeability with increasing feed gas pressure. The pressure at which this trend reverses, that is, corresponding to the minimum permeability, is defined as the plasticization pressure. The olefin permeability in polymer networks, such as PPO, exhibits a sharp multi‐fold rise at a relatively high pressure, which was called “permeability crisis.”^[^ ^145^ ^]^ Simultaneously, the swollen membrane structure leads to a significant deterioration in selectivity. Because of the strong gas solubility in the polymer matrix and high pressure (more than 20 bar for ethylene/ethane separation) used in practical application, there is a conspicuous plasticization effect in olefin/paraffin separation.

**Figure 16:**
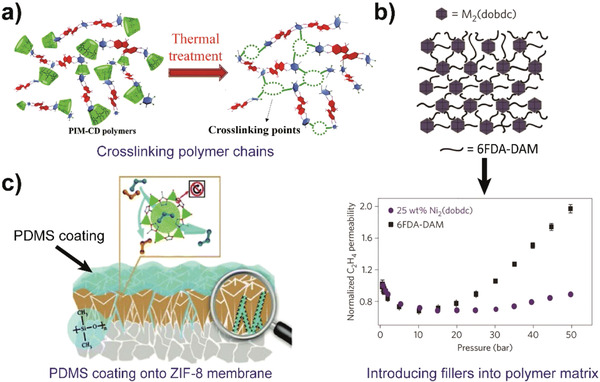
Strategies to inhibit plasticization in membranes. a) Cross‐linking strengthens interchain interaction and mitigates the “swelling” of polymer chains under higher feed pressure. Reproduced with permission.^[^ ^86d^ ^]^ Copyright 2017, Royal Society of Chemistry. b) Introducing fillers could immobilize the polymer chains under high feed pressure and contribute to higher plasticization pressure. Reproduced with permission.^[^ ^23^ ^]^ Copyright 2016, Springer Nature. c) PDMS coating penetrates into the intercrystalline regions and hinders the framework flexibility of ZIF‐8, helping the membrane maintain high selectivity under high pressure difference across the membrane. Reproduced with permission.^[^ ^76^ ^]^ Copyright 2017, Royal Society of Chemistry.

A common strategy to mitigate or suppress plasticization is to strengthen interchain interactions and hinder the movement of polymer chains by cross‐linking. Even though plasticization can be suppressed to some extent, cross‐linking usually entails some loss of permeability because of lower *FFV* and amount of free volume. Liu et al. synthesized *β*‐cyclodextrin in situ cross‐linked PIM‐1 membranes and investigated the membrane structure and separation performance under different annealing temperatures (from 300 to 600 °C).^[^ ^86d^ ^]^ *β*‐cyclodextrin decomposed at high temperatures and generated microvoids in the membrane. Simultaneously, some free radicals that were generated promoted cross‐linking of the PIM‐1 network (**Figure** **16**a). The separation performance varied with the annealing temperature and the high annealing temperature improved plasticization resistance under higher feed gas pressure.

Mixed matrix membranes are also an effective approach, which stabilizes polymer chains through strong interactions between the filler and polymer matrix or cross‐links the polymer. Microporous fillers can introduce additional free volume or transport channels to improve permeability. Bachman et al. utilized 6FDA‐DAM and a series of MOF‐74 as fillers to fabricate mixed matrix membranes, as shown in Figure 16b.^[^ ^23^ ^]^ The coordinatively unsaturated metal sites in MOF‐74 interact with polymer chains and reduce the mobility. The plasticization pressure of the membrane with 25 wt% Ni_2_(dobdc) has a shift from about 10 bar for pristine membrane to 20 bar, demonstrating enhanced anti‐plasticization capability.

Increasing operating temperature also contributes to mitigate the swelling of polymer chains and plasticization because it weakens sorption. Liu et al. increased the operating temperature from 35 to 55 °C and demonstrated that no plasticization occurred at 900 kPa.^[^ ^81^ ^]^

Apart from polymeric membranes, framework structure membranes like ZIF‐8 membranes also experience performance deterioration under high feed gas pressures. For example, Sheng et al. observed that the propylene/propane selectivity of ZIF‐8 membrane decreased from 41 at 1 bar to 8 at 7 bar, mainly because of the gate opening effect at higher pressure.^[^ ^76^ ^]^ Separately, a facile strategy to suppress the framework flexibility and to mitigate plasticization by PDMS coating was proposed (Figure 16c). The propylene/propane selectivity even slightly increased from 93 to 105 as the total transmembrane pressure difference increased from 0 to 6 bar. This abnormal selectivity increase mainly resulted from a sharp decrease of propane permeance. Therefore, enhancing framework stability is helpful to improve plasticization resistance.

#### 4.3.3 Long‐Term Stability

The long‐term stability or aging effect is another aspect of membrane stability. Unlike the deactivation of carriers, the long‐term stability of polymeric membranes, CMS membranes or MOF membranes refers mainly to the stability of free volume or transport channels. Membranes with substantial microporous structures, such as PIMs and CMS membranes, are prone to undergo physical aging. The micropores of PIMs are created from inefficient packing of the rigid chains with contorted sites. These micropores are under non‐thermodynamic equilibrium conditions and tend to pack more efficiently and densely over time, resulting in a decrease of free volume and micropore size. Therefore, the gas permeability of PIM membranes usually decreases quickly at first and tends to be stable over time with the selectivity increasing slightly.

In order to stabilize the membrane structure in PIMs, strengthening interchain interaction is essential and strategies are somewhat similar to plasticization resistance. For CMS membranes, the aging effect is usually related to the irreversible chemical adsorption of atmospheric oxygen and the formation C—O bonds. This increases pore polarity, resulting in more H_2_O molecules being adsorbed. Thus, the pore size is reduced, leading to decreased gas permeability. Moreover, thermal treatment was unable completely recover the gas permeability. This appears to be a significant shortcoming, requiring CMS membranes to be preserved under inert atmosphere. Menendez et al. reported that the permeability loss was only 15–35% for CMS membranes under N_2_ after 30 days, while up to 80% under air exposure.^[^ ^84c^ ^]^

## 5 Conclusions and Outlook

Olefin/paraffin separations are of vital importance to the chemical industry, but established cryogenic fractional distillation processes consume enormous amounts of energy. Membrane technology exhibits great potential in olefin/paraffin separations owing to the merits of thermodynamic and kinetic mechanisms, absence of phase change, and tunable membrane structures. In this review, channel‐based and carrier‐based membranes for ethylene/ethane and propylene/propane separations are summarized. Channel‐based membranes are for the first time sub‐categorized into those with network structures and those with framework structures. The membrane structures, typical membranes, regulation methods, and properties of olefin/paraffin separations, as well as other issues related to practical applications are discussed. Specifically, the manipulation strategies of framework membranes (i.e., zeolite and MOF membranes) toward membrane fabrication, pore size and distribution, grain boundary structures, and framework flexibility are elucidated. The high separation performance of carrier‐based membranes toward ethylene/ethane gas pairs and ZIF‐8 membranes toward propylene/propane gas pairs demonstrates the greater effectiveness of carriers and framework structures. Furthermore, exploration on fabricating thinner membranes and hollow fiber modules, inhibition of plasticization, and aging effect are driving membrane‐based olefin/paraffin closer toward practical applications.

Although significant advances have been realized, there are still significant remaining challenges to overcome before membranes can be adopted for olefin/paraffin separations.

First, an improvement in ethylene/ethane selectivity is a high priority. Apart from some carrier‐based membranes, the ethylene/ethane selectivity of most membranes is less than 10, which is far below practical application requirements. The difference of molecular size between ethylene and ethane is so small that it is difficult to achieve high selectivity without carriers. It is indispensable to integrate different separation mechanisms into the membrane, such as the combination of facilitated transport with molecular sieving, as reported by Dou et al.^[^ ^129^ ^]^ Configurational differences between planar ethylene and spatial ethane could also be exploited by creating shaped membrane pores. Further, exploration of appropriate MOF materials toward ethylene/ethane separation is highly desired. Although, ZIF‐8 membranes exhibit high propylene/propane selectivity, there are currently no known MOF membranes that exhibit high ethylene/ethane selectivity. Encouragingly, the concept of materials genomics has been proposed, which focuses on developing high‐throughput computational tools for material design to construct large materials database. In this endeavor, molecular simulations of ethylene/ethane transport in various MOF materials will be fruitful. The results could provide insights for the development of MOF membranes and verify theoretically whether there is any MOF membrane that could distinguish ethylene from ethane efficiently.

Second, developing membranes that preferentially permeate paraffin is of great interest. For the feed gas containing a small amount of paraffin, it is more economically viable to permeate paraffin rather than olefin. This would allow recovery of the purified olefin to be directly obtained on the retentate side of the membrane module, with less pressure loss. However, there have been no reports about membranes that preferentially permeate paraffin. It is necessary to overcome the faster diffusion of olefins and to minimize the diffusion selectivity of olefin/paraffin. Specific carriers or new separation mechanisms are needed. In recent years, adsorbents which preferentially adsorb paraffins over olefins have appeared, such as Fe_2_(O_2_)(dobdc),^[^ ^104^ ^]^ MAF‐49,^[^ ^146^ ^]^ IRMOF‐8,^[^ ^147^ ^]^ and PCN‐245.^[^ ^148^ ^]^ In addition, in O_2_/N_2_ separation and N_2_/CH_4_ separation, membranes that preferentially permeate larger molecules have been reported.^[^ ^149^ ^]^ The host–guest interactions and mass transport mechanisms in these adsorbents or membranes can guide the development of membranes that preferentially permeate paraffins.

Third, the development of new kinds of membranes with framework structures, such as covalent organic frameworks (COFs), which possess covalently linked topology, is of rapidly growing interest. Their ordered crystalline framework structures, inter‐connective pores with controllable size and functionalization, as well as the framework tenability endow COFs with great potential in membrane separation. The transition from purely inorganic zeolite to inorganic–organic hybrid MOFs and then to purely organic COFs, reflects a new trend for the development of membrane materials. Also, the rigid covalent frameworks endow COF membranes with excellent tolerance and stability under harsh conditions, which may be helpful to anti‐plasticization and anti‐aging. There have been several reports about COF membranes in water treatment, pervaporation, hydrogen purification, and carbon capture. However, preparing defect‐free COF membranes is still challenging, and currently, the pore size of COF membranes is often too large for ethylene/ethane and propylene/propane separation. Moreover, more control in fabricating COF membranes with appropriate pore size is needed for olefin/paraffin separations.

Fourth, improving the membrane stability under actual conditions is an important consideration for practical adoption. High pressure feeds and impurities such as hydrogen sulfide, which is extremely harmful to the carriers of metal ions, are difficult challenges. Currently, there are few effective measures to protect carriers from poisoning; so simple and effective strategies are needed to inhibit the deactivation of metal ions. For the plasticization and aging resistance, hyper‐crosslinking may possibly address this issue for polymeric membranes because it creates strong interchain interactions without sacrificing free volume. This strategy could also create more *FFV* to improve gas permeability.

Fifth, developing large‐scale fabrication methods for MOF membranes, especially with hollow fiber configuration, is crucial for industrial adoption. Hollow fiber membranes are possibly the most promising for commercial or practical application compared with other configurations because of the extremely high membrane areas, large productivity, and low footprint. Moreover, growing MOF layers on the bore side of hollow fibers is more attractive than the shell side because it is easier to maintain the mechanical robustness of the relatively fragile polycrystalline membrane during high density fiber packing into the modules. ZIF‐8 membranes are currently the most promising membranes for practical propylene/propane separation. Until now, various supports (polymeric and ceramic supports) and different fabrication methods (interfacial microfluidic and secondary growth) have been explored toward ZIF‐8‐based hollow fiber membranes. Ultrathin MOF layers with high gas permeance is a credible goal, but still a current challenge.

In conclusion, the future directions on membrane‐based olefin/paraffin separations should be focused on learning from advanced structures in the nature, developing organic molecular sieve membranes, which are constructed by organic building blocks and possess long‐range ordered microporous structures, and integrating multiple mass transport mechanisms. With the rapid development of material genomics, dynamic covalent chemistry, and bionic engineering, we believe that organic molecular sieve membranes, especially COF membranes, with suitable pore size, controllable assembly, and stable facilitated transport carriers will be constructed and be applied to address efficient olefin/paraffin separations.

## Conflict of Interest

The authors declare no conflict of interest.

## Supporting information

Supporting Information
