# Supplementary material for: Membrane‐Based Olefin/Paraffin Separations
Source: Adv Sci (Weinh). 2020 Aug 9;7(19):2001398. doi: 10.1002/advs.202001398 (PMC7539199; doi:10.1002/advs.202001398)
Supplement: Supplementary file 1 — Supporting Information [file ADVS-7-2001398-s001.pdf]

© 2020 Wiley-VCH GmbH

ADVANCED SCIENCE
Open Access

Supporting Information

for *Adv. Sci.,* DOI: 10.1002/advs.202001398

Membrane-Based Olefin/Paraffin Separations

Yanxiong Ren, Xu Liang, Haozhen Dou, Chumei Ye, Zheyuan Guo, Jianyu Wang, Yichang Pan, Hong Wu, Michael D. Guiver,* and Zhongyi Jiang**

Supporting Information

Membrane-based olefin/paraffin separations

Yanxiong Ren, Xu Liang, Haozhen Dou, Chumei Ye, Zheyuan Guo, Jianyu Wang, Yichang Pan, Hong Wu, Michael D. Guiver,* and Zhongyi Jiang**

Y. Ren, X. Liang, H. Dou, C. Ye, Z. Guo, J. Wang, Prof. H. Wu, Prof. Z. Jiang
Key Laboratory for Green Chemical Technology of Ministry of Education
School of Chemical Engineering and Technology
Tianjin University
Tianjin 300072, P.R. China
E-mail: wuhong@tju.edu.cn; zhyjiang@tju.edu.cn

Y. Ren, X. Liang, C. Ye, Z. Guo, J. Wang, Prof. H. Wu, Prof. M. D. Guiver, Prof. Z. Jiang
Collaborative Innovation Center of Chemical Science and Engineering (Tianjin)
Tianjin 300072, China
E-mail: michael.guiver@outlook.com

Prof. M. D. Guiver
State Key Laboratory of Engines
Tianjin University
Tianjin 300072, P.R. China

Prof. Z. Jiang
Joint School of National University of Singapore and Tianjin University
International Campus of Tianjin University
Binhai New City, Fuzhou 350207, P. R. China

Prof. Y. Pan
State Key Laboratory of Materials-Oriented Chemical Engineering
College of Chemical Engineering
Nanjing Tech University
Nanjing 210009, P.R. China

Table S1. Membrane separation performance data of C_2H_4/C_2H_6

Material	Permeability/ Barrer	Selectivity	Membrane Type	Material sub-type	Reference
6FDA-PI	2.1	4.4	Membranes with network structures	Polymers	1
PEO	3	1.76	Membranes with network structures	Polymers	2
Pebax 2533	130	1.4	Membranes with network structures	Polymers	3
poly(2,6-dimethyl-1,4-phenyleneoxide)	160	5.3	Membranes with network structures	Polymers	4
poly(2,6-dimethyl-1,4_phenyleneoxide)97.5/poly(2,6-diphenyl-1,4-phenyleneoxide)2.5	72	2.9	Membranes with network structures	Polymers	4
poly(2,6-dimethyl-1,4_phenyleneoxide)75/poly(2,6-diphenyl-1,4-phenyleneoxide)25	58	4.5	Membranes with network structures	Polymers	4
PETP	0.9	1.8	Membranes with network structures	Polymers	4
Polyethylene	160	1.1	Membranes with network structures	Polymers	4
PTFE	4.9	1.5	Membranes with network structures	Polymers	4
Polysiloxane	30000	1	Membranes with network structures	Polymers	4
6FDA–mPD	0.3	3.3	Membranes with network structures	Polymers	5
6FDA–IPDA	1.4	3.8	Membranes with network structures	Polymers	5
6FDA-1,5-NDA	0.87	5.8	Membranes with network structures	Polymers	6
Ag-Na-X	945	15.9	Membranes with framework structures	Zeolites	7
ZIF-8	1350	2.2	Membranes with framework structures	MOFs	8
ZIF-8	1050	2.3	Membranes with framework structures	MOFs	9
Phenolic resin carbon membrane	330	4.5	Other kinds of channel-based membranes	Carbon membranes	10
Phenolic resin carbon membrane	16	5.4	Other kinds of channel-based membranes	Carbon membranes	11
Sulfonated PI-based carbon membranes	31	4.2	Other kinds of channel-based membranes	Carbon membranes	12
PI-based carbon membrane	10	12	Other kinds of channel-based membranes	Carbon membranes	13
PI-based carbon membrane	16.7	11.7	Other kinds of channel-based membranes	Carbon membranes	14
PIM-1-based carbon membrane	1.3	13	Other kinds of channel-based membranes	Carbon membranes	15
PIM-6FDA-OH carbon membrane	10	17.5	Other kinds of channel-based membranes	Carbon membranes	16
PIM-1-based carbon membrane	13.7	9.7	Other kinds of channel-based membranes	Carbon membranes	17

PIM-based carbon membrane	3	25	Other kinds of channel-based membranes	Carbon membranes	18
PI-based carbon membrane	100	8.53	Other kinds of channel-based membranes	Carbon membranes	19
C_{60}/PS	0.82	1.7	Other kinds of channel-based membranes	Mixed Matrix Membranes	20
CA/SiO_2	0.11	4.07	Other kinds of channel-based membranes	Mixed Matrix Membranes	21
P84/CuBTC	0.051	6.88	Other kinds of channel-based membranes	Mixed Matrix Membranes	22
6FDA-DAM/MOF-74	320	4.6	Other kinds of channel-based membranes	Mixed Matrix Membranes	23
Fluorinated PI/TiO_2	0.095	2.67	Other kinds of channel-based membranes	Mixed Matrix Membranes	24
Matrimid/SiO_2	0.411	6.13	Other kinds of channel-based membranes	Mixed Matrix Membranes	25
P84/MOF	0.051	7.1	Other kinds of channel-based membranes	Mixed Matrix Membranes	26
PPEES/ZIF-8	3.15	3	Other kinds of channel-based membranes	Mixed Matrix Membranes	27
CuCl+Eutectic solvent	122.2	12.5	Carrier-based membranes	-	28
IL+Ag	13.1	7.24	Carrier-based membranes	-	29
GO+IL+Ag^+	145	215	Carrier-based membranes	-	30
BN+IL+Ag^+	27.6	128	Carrier-based membranes	-	31
PEO+$AgBF_4$	40	240	Carrier-based membranes	-	32
DES/CuCl	13	19	Carrier-based membranes	-	33
PIL+$AgNO_3$	80	42	Carrier-based membranes	-	34

Table S2. Membrane separation performance data of C_3H_6/C_3H_8

Material	Permeability/ Barrer	Selectivity	Membrane type	Material sub-type	Reference
poly(2,6-dimethyl-1,4-phenyleneoxide)	116	17.8	Membranes with network structures	Polymers	4
6FDA-TeMPD	37	8.6	Membranes with network structures	Polymers	35
6FDA-TrMPD	30	11	Membranes with network structures	Polymers	35
6FDA-DDBT	0.76	27	Membranes with network structures	Polymers	35
6FDA-ODA	0.48	11	Membranes with network structures	Polymers	35
BPDA-TeMPD	3.2	13	Membranes with network structures	Polymers	35
PPO	2.9	9.1	Membranes with network structures	Polymers	35
P4MP	54	2	Membranes with network structures	Polymers	35
1.2PB	260	1.7	Membranes with network structures	Polymers	35
PDMS	6600	1.1	Membranes with network structures	Polymers	35
Matrimid® 5218	0.1	16	Membranes with network structures	Polymers	36
6FDA-1,5-NDA	0.24	5.8	Membranes with network structures	Polymers	6
6FDA-BPDA/DDBT(1/1)	1	31	Membranes with network structures	Polymers	37
PEG-biazide crosslinked PIM	1051	7.5	Membranes with network structures	Polymers	38
PIM-PIs	393	6	Membranes with network structures	Polymers	39
6FDA-DAM	15.7	12.4	Membranes with network structures	Polymers	40
sulfonylated PPO	18.1	3.05	Membranes with network structures	Polymers	41
6FDA-based PI	0.89	16	Membranes with network structures	Polymers	1
PEO	6.6	2.75	Membranes with network structures	Polymers	2
P84/Matrimid	7	18.2	Membranes with network structures	Polymers	42
PI-β-CD	81.4	13.88	Membranes with network structures	Polymers	43
PIM-PI	817	16	Membranes with network structures	Polymers	44
azide-PIM	40	10	Membranes with network structures	Polymers	45
crosslinked PI	4.5	11	Membranes with network structures	Polymers	46
Pebax 2533	580	1.6	Membranes with network structures	Polymers	3
Na-X-Ag	420	50	Membranes with framework structures	Zeolites	7
ETS-10	1920	5.5	Membranes with framework structures	Zeolites	47
Zeolite Y-Ag	1016	4.8	Membranes with framework structures	Zeolites	48
Na-X	520	6.2	Membranes with framework structures	Zeolites	49
Na-X	428	13.7	Membranes with framework structures	Zeolites	50
Na-X	420	3.3	Membranes with framework structures	Zeolites	51
Zn-ZIF-67	66	50.5	Membranes with	MOFs	52

			framework structures		
ZIF-8	136	45	Membranes with framework structures	MOFs	53
ZIF-8	9	110	Membranes with framework structures	MOFs	54
ZIF-8	90	50	Membranes with framework structures	MOFs	55
ZIF-8	93.6	40	Membranes with framework structures	MOFs	56
ZIF-8	82.5	30	Membranes with framework structures	MOFs	57
ZIF-8@ZIF-67	370	209	Membranes with framework structures	MOFs	58
ZIF-8	80.4	70	Membranes with framework structures	MOFs	59
ZIF-8	10.7	150	Membranes with framework structures	MOFs	60
ZIF-8	340.2	180	Membranes with framework structures	MOFs	61
ZIF-8	330	65	Membranes with framework structures	MOFs	62
ZIF-8	45	27.8	Membranes with framework structures	MOFs	63
ZIF-8	37.2	61	Membranes with framework structures	MOFs	64
ZIF-8	255	36	Membranes with framework structures	MOFs	65
ZIF-8	73	70	Membranes with framework structures	MOFs	66
ZIF-8	25	79	Membranes with framework structures	MOFs	67
ZIF-8	67.2	80	Membranes with framework structures	MOFs	68
ZIF-8	10.4	304.8	Membranes with framework structures	MOFs	69
ZIF-8	24.75	139.3	Membranes with framework structures	MOFs	70
ZIF-8	44	46	Membranes with framework structures	MOFs	71
ZIF-8	148.5	31.6	Membranes with framework structures	MOFs	72
ZIF-8	81	105	Membranes with framework structures	MOFs	73
ZIF-8	180	191	Membranes with framework structures	MOFs	74
ZIF-8	41	55	Membranes with framework structures	MOFs	75
ZIF-8	178	50	Membranes with framework structures	MOFs	76
ZIF-8	38	94	Membranes with framework structures	MOFs	77
ZIF-8	53.1	27	Membranes with framework structures	MOFs	78
ZIF-8	48	90.2	Membranes with framework structures	MOFs	79
ZIF-8	91	142	Membranes with framework structures	MOFs	80
Phenolic resin carbon membrane	90	17	Other kinds of channel-based membranes	Carbon Membranes	81
Phenolic resin carbon membrane	200	3.1	Other kinds of channel-based membranes	Carbon Membranes	10
PI-based carbon membrane	5.2	22	Other kinds of channel-based membranes	Carbon Membranes	82
Phenolic resin carbon membrane	19	5.4	Other kinds of channel-based membranes	Carbon Membranes	11

Sulfonated PI-based carbon membranes	15	4.2	Other kinds of channel-based membranes	Carbon Membranes	12
Interpenetrating network-based carbon membrane	48	44	Other kinds of channel-based membranes	Carbon Membranes	83
PI-based carbon membrane	14.4	36	Other kinds of channel-based membranes	Carbon Membranes	84
Silicon membrane	13.3	37	Other kinds of channel-based membranes	Carbon Membranes	85
PI-based carbon membrane	0.528	35	Other kinds of channel-based membranes	Carbon Membranes	86
PIM-PI-based carbon membrane	45	33	Other kinds of channel-based membranes	Carbon Membranes	87
PI-based carbon membrane	15.6	31	Other kinds of channel-based membranes	Carbon Membranes	88
PIM-β-CD	1500	5.8	Other kinds of channel-based membranes	Carbon Membranes	89
PI-based carbon membrane	240	30	Other kinds of channel-based membranes	Carbon Membranes	90
PI-based carbon membrane	403	25	Other kinds of channel-based membranes	Carbon Membranes	91
6FDA-DAM/ZIF-8	100	6.6	Other kinds of channel-based membranes	Mixed Matrix Membranes	92
Ethy CA/C_{60}	61.3	4.9	Other kinds of channel-based membranes	Mixed Matrix Membranes	93
CA/SiO_2	0.098	6.12	Other kinds of channel-based membranes	Mixed Matrix Membranes	21
Fluorinated PI/TiO_2	0.08	5.11	Other kinds of channel-based membranes	Mixed Matrix Membranes	24
Matrimid/SiO_2	0.16	18.03	Other kinds of channel-based membranes	Mixed Matrix Membranes	25
Thermal crosslinked PI/ZIF-8	42.7	27.47	Other kinds of channel-based membranes	Mixed Matrix Membranes	94
6FDA-DAM+Y-fum-fcu-MOF	33.4	18	Other kinds of channel-based membranes	Mixed Matrix Membranes	95
PIM-6FDA-OH/ZIF-8	38	43	Other kinds of channel-based membranes	Mixed Matrix Membranes	96
MFI/ZIF-8	548	146	Other kinds of channel-based membranes	Mixed Matrix Membranes	97
XLPEO/ZIF-8	28	15	Other kinds of channel-based membranes	Mixed Matrix Membranes	98
PU/ZIF-8	140	3.5	Other kinds of channel-based membranes	Mixed Matrix Membranes	99
SBS/Cu@MIL-101(Cr)	353	1.9	Other kinds of channel-based membranes	Mixed Matrix Membranes	100
Pebax 1657/ZIF-8	84	5.57	Other kinds of channel-based membranes	Mixed Matrix Membranes	101
6FDA-DAM/ZIF-8	56.2	31	Other kinds of channel-based membranes	Mixed Matrix Membranes	102
6FDA-DAM/ZIF-67	34.1	29.9	Other kinds of channel-based membranes	Mixed Matrix Membranes	103

PIM-1/SIFSIX-3-Zn	4012	7.9	Other kinds of channel-based membranes	Mixed Matrix Membranes	104
Ag nanoparticles	5	170	Carrier-based membranes	-	105
PVP-$AgBF_4$	7.5	155	Carrier-based membranes	-	106
POZ-$AgBF_4$	15	135	Carrier-based membranes	-	107
PI-$AgBF_4$	0.3	6.6	Carrier-based membranes	-	108
PPO-Metal ions	12.5	5.2	Carrier-based membranes	-	109
PAAm-$AgBF_4$	480	6.8	Carrier-based membranes	-	110
PEP-$AgBF_4$	6	55	Carrier-based membranes	-	111
POZ-Ag	5	100	Carrier-based membranes	-	112
POZ-$AgNO_3$-SiO_2	1.02	88	Carrier-based membranes	-	113
Au-DMAP-PVP/PSf	6	22	Carrier-based membranes	-	114
POZ-$AgBF_4$-$Al(NO_3)_3$	4.8	21	Carrier-based membranes	-	115
$Al_2O_3(SiO_2)$-$AgNO_3$	51	7.7	Carrier-based membranes	-	116
PEO-$AgBF_4$	72	1000	Carrier-based membranes	-	117
PVDF-HFP-$AgBF_4$	1500	300	Carrier-based membranes	-	118

References

[1] C. Staudt-Bickel, W. J. Koros, J. Membr. Sci. **2000**, *170*, 205.

[2] H. Lin, B. D. Freeman, J. Membr. Sci. **2004**, *239*, 105.

[3] J. C. Chen, X. S. Feng, A. Penlidis, Sep. Sci. Technol. **2004**, *39*, 149.

[4] O. M. Ilinitch, G. L. Semin, M. V. Chertova, K. I. Zamaraev, J. Membr. Sci. **1992**, *66*, 1.

[5] C. Staudt-Bickel, W. J. Koros, J. Membr. Sci. **2000**, *170*, 205.

[6] S. S. Chan, R. Wang, T. S. Chung, Y. Liu, J. Membr. Sci. **2002**, *210*, 55.

[7] M. Sakai, Y. Sasaki, T. Tomono, M. Seshimo, M. Matsukata, ACS Appl. Mater. Interfaces **2019**, *11*, 4145.

[8] H. Bux, C. Chmelik, R. Krishna, J. Caro, J. Membr. Sci. **2011**, *369*, 284.

[9] J. B. James, J. Wang, L. Meng, Y. S. Lin, Ind. Eng. Chem. Res. **2017**, *56*, 7567.

[10] A. B. Fuertes, I. Menendez, Sep. Purif. Technol. **2002**, *28*, 29.

[11] T. A. Centeno, J. L. Vilas, A. B. Fuertes, J. Membr. Sci. **2004**, *228*, 45.

[12] M. N. Islam, W. Zhou, T. Honda, K. Tanaka, H. Kita, K.-i. Okamoto, J. Membr. Sci. **2005**, *261*, 17.

[13] L. Xu, M. Rungta, W. J. Koros, J. Membr. Sci. **2011**, *380*, 138.

[14] M. Rungta, L. Xu, W. J. Koros, Carbon **2012**, *50*, 1488.

[15] O. Salinas, X. Ma, E. Litwiller, I. Pinnau, J. Membr. Sci. **2016**, *504*, 133.

[16] O. Salinas, X. Ma, E. Litwiller, I. Pinnau, J. Membr. Sci. **2016**, *500*, 115.

[17] K.-S. Liao, S. Japip, J.-Y. Lai, T.-S. Chung, J. Membr. Sci. **2017**, *534*, 92.

[18] O. Salinas, X. Ma, Y. Wang, Y. Han, I. Pinnau, RSC Adv. **2017**, *7*, 3265.

[19] Y.-H. Chu, D. Yancey, L. Xu, M. Martinez, M. Brayden, W. Koros, J. Membr. Sci. **2018**, *548*, 609.

[20] A. Higuchi, T. Agatsuma, S. Uemiya, T. Kojima, K. Mizoguchi, I. Pinnau, K. Nagai, B. D. Freeman, J. Appl. Polym. Sci. **2000**, *77*, 529.

[21] M. Naghsh, M. Sadeghi, A. Moheb, M. P. Chenar, M. Mohagheghian, J. Membr. Sci. **2012**, *423*, 97.

[22] J. Ploegmakers, S. Japip, K. Nijmeijer, J. Membr. Sci. **2013**, *428*, 331.

[23] J. E. Bachman, Z. P. Smith, T. Li, T. Xu, J. R. Long, Nat. Mater. **2016**, *15*, 845.

[24] H. Ahmadizadegan, F. Ghavvas, M. Ranjbar, S. Esmaielzadeh, Polym. Bull. **2018**, *75*, 2729.

[25] S. M. Davoodi, M. Sadeghi, M. Naghsh, A. Moheb, RSC Adv. **2016**, *6*, 23746.

[26] J. Ploegmakers, S. Japip, K. Nijmeijer, J. Membr. Sci. **2013**, *428*, 445.

[27] K. Diaz, M. Lopez-Gonzalez, L. F. del Castillo, E. Riande, J. Membr. Sci. **2011**, *383*, 206.

[28] R. Deng, Y. Sun, H. Bi, H. Dou, H. Yang, B. Wang, W. Tao, B. Jiang, Energy Fuels **2017**, *31*, 11146.

[29] L. C. Tome, D. Mecerreyes, C. S. R. Freire, L. P. N. Rebelo, I. M. Marrucho, J. Mater. Chem. A **2014**, *2*, 5631.

[30] H. Dou, M. Xu, B. Jiang, G. Wen, L. Zhao, B. Wang, A. Yu, Z. Bai, Y. Sun, L. Zhang, Z. Chen, Z. Jiang, Adv. Funct. Mater. **2019**, *29*, 1905229.

[31] H. Dou, B. Jiang, M. Xu, Z. Zhang, G. Wen, F. Peng, A. Yu, Z. Bai, Y. Sun, L. Zhang, Z. Jiang, Z. Chen, Angew. Chem., Int. Ed. **2019**, *58*, 13969.

[32] I. Pinnau, L. G. Toy, J. Membr. Sci. **2001**, *184*, 39.

[33] B. Jiang, H. Dou, L. Zhang, B. Wang, Y. Sun, H. Yang, Z. Huang, H. Bi, J. Membr. Sci. **2017**, *536*, 123.

[34] H. Dou, B. Jiang, X. Xiao, M. Xu, B. Wang, L. Hao, Y. Sun, L. Zhang, J. Membr. Sci. **2018**, *557*, 76.

[35] K. Tanaka, A. Taguchi, J. Hao, H. Kita, K. Okamoto, J. Membr. Sci. **1996**, *121*, 197.

[36] J. J. Krol, M. Boerrigter, G. H. Koops, J. Membr. Sci. **2001**, *184*, 275.

[37] M. Yoshino, S. Nakamura, H. Kita, K.-i. Okamoto, N. Tanihara, Y. Kusuki, J. Membr. Sci. **2003**, *212*, 13.

[38] M. M. Khan, G. Bengtson, S. Shishatskiy, B. N. Gacal, M. Mushfequr Rahman, S. Neumann, V. Filiz, V. Abetz, Eur. Polym. J. **2013**, *49*, 4157.

[39] R. J. Swaidan, B. Ghanem, R. Swaidan, E. Litwiller, I. Pinnau, J. Membr. Sci. **2015**, *492*, 116.

[40] C. Zhang, Y. Dai, J. R. Johnson, O. Karvan, W. J. Koros, J. Membr. Sci. **2012**, *389*, 34.

[41] S. B. Gajbhiye, Indian J. Chem. Technol. **2015**, *22*, 105.

[42] H. Lin, B. D. Freeman, J. Membr. Sci. **2004**, *239*, 105.

[43] M. Askari, T. Yang, T.-S. Chung, J. Membr. Sci. **2012**, *423*, 392.

[44] R. J. Swaidan, B. Ghanem, R. Swaidan, E. Litwiller, I. Pinnau, J. Membr. Sci. **2015**, *492*, 116.

[45] N. Du, M. M. Dal-Cin, I. Pinnau, A. Nicalek, G. P. Robertson, M. D. Guiver, Macromol. Rapid Commun. **2011**, *32*, 631.

[46] C. Staudt-Bickel, Soft Materials **2003**, *1*, 277.

[47] I. Tiscornia, S. Irusta, C. Téllez, J. Coronas, J. Santamaría, J. Membr. Sci. **2008**, *311*, 326.

[48] S. Shrestha, P. K. Dutta, Microporous Mesoporous Mater. **2019**, *279*, 178.

[49] V. Nikolakis, G. Xomeritakis, A. Abibi, M. Dickson, M. Tsapatsis, D. G. Vlachos, J. Membr. Sci. **2001**, *184*, 209.

[50] I. G. Giannakopoulos, V. Nikolakis, Ind. Eng. Chem. Res. **2005**, *44*, 226.

[51] A. Mundstock, N. Wang, S. Friebe, J. Caro, Microporous Mesoporous Mater. **2015**, *215*, 20.

[52] C. Wang, F. Yang, L. Sheng, J. Yu, K. Yao, L. Zhang, Y. Pan, Chem. Commun. **2016**, *52*, 12578.

[53] Y. Pan, T. Li, G. Lestari, Z. Lai, J. Membr. Sci. **2012**, *390-391*, 93.

[54] X. Ma, P. Kumar, N. Mittal, A. Khlyustova, P. Daoutidis, K. A. Mkhoyan, M. Tsapatsis, Science **2018**, *361*, 1008.

[55] H. T. Kwon, H.-K. Jeong, J. Am. Chem. Soc. **2013**, *135*, 10763.

[56] H. T. Kwon, H.-K. Jeong, Chem. Commun. **2013**, *49*, 3854.

[57] D. Liu, X. Ma, H. Xi, Y. S. Lin, J. Membr. Sci. **2014**, *451*, 85.

[58] H. T. Kwon, H.-K. Jeong, A. S. Lee, H. S. An, J. S. Lee, J. Am. Chem. Soc. **2015**, *137*, 12304.

[59] H. T. Kwona, H.-K. Jeong, Chem. Eng. Sci. **2015**, *124*, 20.

[60] E. Barankova, X. Tan, L. F. Villalobos, E. Litwiller, K.-V. Peinemann, Angew. Chem., Int. Ed. **2017**, *56*, 2965.

[61] K. Eum, C. Ma, A. Rownaghi, C. W. Jones, S. Nair, ACS Appl. Mater. Interfaces **2016**, *8*, 25337.

[62] K. Eum, A. Rownaghi, D. Choi, R. R. Bhave, C. W. Jones, S. Nair, Adv. Funct. Mater. **2016**, *26*, 5011.

[63] E. Shamsaei, X. Lin, Z.-X. Low, Z. Abbasi, Y. Hu, J. Z. Liu, H. Wang, ACS Appl. Mater. Interfaces **2016**, *8*, 6236.

[64] J. Yu, Y. Pan, C. Wang, Z. Lai, Chem. Eng. Sci. **2016**, *141*, 119.

[65] S. Tanaka, K. Okubo, K. Kida, M. Sugita, T. Takewaki, J. Membr. Sci. **2017**, *544*, 306.

[66] W. Li, P. Su, Z. Li, Z. Xu, F. Wang, H. Ou, J. Zhang, G. Zhang, E. Zeng, Nat. Commun. **2017**, *8*.

[67] L. Sheng, C. Wang, F. Yang, L. Xiang, X. Huang, J. Yu, L. Zhang, Y. Pan, Y. Li, Chem. Commun. **2017**, *53*, 7760.

[68] J. Sun, C. Yu, H.-K. Jeong, Crystals **2018**, *8*.

[69] S. Zhou, Y. Wei, L. Li, Y. Duan, Q. Hou, L. Zhang, L.-X. Ding, J. Xue, H. Wang, J. Caro, Sci. Adv. **2018**, *4*, eaau1393.

[70] K. Huang, B. Wang, Y. Chi, K. Li, Adv. Mater. Interfaces **2018**, *5*.

[71] M. J. Lee, M. R. A. Hamid, J. Lee, J. S. Kim, Y. M. Lee, H.-K. Jeong, J. Membr. Sci. **2018**, *559*, 28.

[72] G. He, M. Dakhchoune, J. Zhao, S. Huang, K. V. Agrawal, Adv. Funct. Mater. **2018**, *28*, 1707427.

[73] G. Ramu, M. Lee, H.-K. Jeong, Microporous Mesoporous Mater. **2018**, *259*, 155.

[74] W. Li, W. Wu, Z. Li, J. Shi, Y. Xia, J. Mater. Chem. A **2018**, *6*, 16333.

[75] M. R. Abdul Hamid, S. Park, J. S. Kim, Y. M. Lee, H.-K. Jeong, Ind. Eng. Chem. Res. **2019**, *58*, 14947.

[76] F. Hillman, H.-K. Jeong, ACS Appl. Mater. Interfaces **2019**, *11*, 18377.

[77] X. Jiang, S. Li, Y. Bai, L. Shao, J. Mater. Chem. A **2019**, *7*, 10898.

[78] M. R. A. Hamid, S. Park, J. S. Kim, Y. M. Lee, H.-K. Jeong, J. Mater. Chem. A **2019**, *7*, 9680.

[79] J. H. Lee, D. Kim, H. Shin, S. J. Yoo, H. T. Kwon, J. Kim, J. Ind. Eng. Chem. **2019**, *72*, 374.

[80] R. Wei, H.-Y. Chi, X. Li, D. Lu, Y. Wan, C.-W. Yang, Z. Lai, Adv. Funct. Mater. **2020**, *30*, 1907089.

[81] I. Menendez, A. B. Fuertes, Carbon **2001**, *39*, 733.

[82] M. Yoshino, S. Nakamura, H. Kita, K.-i. Okamoto, N. Tanihara, Y. Kusuki, J. Membr. Sci. **2003**, *215*, 169.

[83] M. L. Chng, Y. Xiao, T.-S. Chung, M. Toriida, S. Tamai, Carbon **2009**, *47*, 1857.

[84] X. Ma, B. K. Lin, X. Wei, J. Kniep, Y. S. Lin, Ind. Eng. Chem. Res. **2013**, *52*, 4297.

[85] S. M. Ibrahim, R. Xu, H. Nagasawa, A. Naka, J. Ohshita, T. Yoshioka, M. Kanezashi, T. Tsuru, RSC Adv. **2014**, *4*, 12404.

[86] X. Ma, S. Williams, X. Wei, J. Kniep, Y. S. Lin, Ind. Eng. Chem. Res. **2015**, *54*, 9824.

[87] R. J. Swaidan, X. Ma, I. Pinnau, J. Membr. Sci. **2016**, *520*, 983.

[88] X. Ma, Y. S. Lin, X. Wei, J. Kniep, AIChE J. **2016**, *62*, 491.

[89] J. Liu, Y. Xiao, T.-S. Chung, J. Mater. Chem. A **2017**, *5*, 4583.

[90] S.-J. Kim, P. S. Lee, J.-S. Chang, S.-E. Nam, Y.-I. Park, Sep. Purif. Technol. **2018**, *194*, 443.

[91] C. Karunaweera, I. H. Musselman, K. J. Balkus, Jr., J. P. Ferraris, J. Membr. Sci. **2019**, *581*, 430.

[92] C. Zhang, K. Zhang, L. Xu, Y. Labreche, B. Kraftschik, W. J. Koros, AIChE J. **2014**, *60*, 2625.

[93] H. Sun, C. Ma, T. Wang, Y. Xu, B. Yuan, P. Li, Y. Kong, Chem. Eng. Technol. **2014**, *37*, 611.

[94] M. Askari, T.-S. Chung, J. Membr. Sci. **2013**, *444*, 173.

[95] Y. Liu, Z. Chen, G. Liu, Y. Belmabkhout, K. Adil, M. Eddaoudi, W. Koros, Adv. Mater. **2019**, *31*, 1807513.

[96] X. Ma, R. J. Swaidan, Y. Wang, C.-e. Hsiung, Y. Han, I. Pinnau, ACS Appl. Nano Mater. **2018**, *1*, 3541.

[97] F. Rashidi, J. Leisen, S.-J. Kim, A. A. Rownaghi, C. W. Jones, S. Nair, Angew. Chem., Int. Ed. **2019**, *58*, 236.

[98] D. Liu, L. Xiang, H. Chang, K. Chen, C. Wang, Y. Pan, Y. Li, Z. Jiang, Chem. Eng. Sci. **2019**, *204*, 151.

[99] H. R. Amedi, M. Aghajani, Chem. Pap. **2018**, *72*, 1477.

[100] J. P. Jung, M. J. Kim, Y.-S. Bae, J. H. Kim, J. Appl. Polym. Sci. **2018**, *135*.

[101] H. R. Amedi, M. Aghajani, J. Appl. Polym. Sci. **2018**, *135*.

[102] C. Zhang, Y. Dai, J. R. Johnson, O. Karvan, W. J. Koros, J. Membr. Sci. **2012**, *389*, 34.

[103] H. An, S. Park, H. T. Kwon, H.-K. Jeong, J. S. Lee, J. Membr. Sci. **2017**, *526*, 367.

[104] Q. Shen, S. Cong, R. He, Z. Wang, Y. Jin, H. Li, X. Cao, J. Wang, B. Van der Bruggen, Y. Zhang, J. Membr. Sci. **2019**, *588*.

[105] Y. S. Kang, S. W. Kang, H. Kim, J. H. Kim, J. Won, C. K. Kim, K. Char, Adv. Mater. **2007**, *19*, 475.

[106] B. Jose, J. H. Ryu, B. G. Lee, H. Lee, Y. S. Kang, H. S. Kim, Chem. Commun. **2001**, 2046.

[107] B. Jose, J. H. Ryu, Y. J. Kim, H. Kim, Y. S. Kang, S. D. Lee, H. S. Kim, Chem. Mater. **2002**, *14*, 2134.

[108] S. Hess, G. Scharfenberger, C. Staudt-Bickel, R. N. Lichtenthaler, Desalination **2002**, *145*, 359.

[109] S. Bai, S. Sridhar, A. A. Khan, J. Membr. Sci. **1998**, *147*, 131.

[110] Y. S. Park, J. Won, Y. S. Kang, J. Membr. Sci. **2001**, *183*, 163.

[111] S. W. Kang, J. H. Kim, K. S. Oh, J. Won, K. Char, H. S. Kim, Y. S. Kang, J. Membr. Sci. **2004**, *236*, 163.

[112] J. H. Kim, S. M. Park, J. Won, Y. S. Kang, J. Membr. Sci. **2005**, *248*, 171.

[113] S. W. Kang, J. H. Kim, K. Char, J. Won, Y. S. Kang, J. Membr. Sci. **2006**, *285*, 102.

[114] S. W. Kang, J. Hong, J. H. Park, S. H. Mun, J. H. Kim, J. Cho, K. Char, Y. S. Kang, J. Membr. Sci. **2008**, *321*, 90.

[115] S. W. Kang, J. H. Kim, J. Won, Y. S. Kang, J. Membr. Sci. **2013**, *445*, 156.

[116] K. A. Stoitsas, A. Gotzias, E. S. Kikkinides, T. A. Steriotis, N. K. Kanellopoulos, M. Stoukides, V. T. Zaspalis, Microporous Mesoporous Mater. **2005**, *78*, 235.

[117] L. Liu, X. S. Feng, A. Chakma, Sep. Purif. Technol. **2004**, *38*, 255.

[118] R. Zarca, A. Ortiz, D. Gorri, I. Ortiz, Sep. Purif. Technol. **2017**, *180*, 82.
